# Clinical characterization of acute COVID-19 and Post-COVID-19 Conditions 3 months following infection: A cohort study among Indigenous adults and children in the Southwestern United States

**DOI:** 10.1371/journal.pgph.0004204

**Published:** 2025-03-18

**Authors:** Chelsea S. Lutz, Rachel M. Hartman, Marqia Sandoval, Amanda B. Burrage, Loretta Christensen, Ryan M. Close, Shawnell Damon, Tarayn A. Fairlie, Melissa B. Hagen, Alexa M. Kugler, Oliver Laeyendecker, Elvira Honie, Verlena Little, Heba H. Mostafa, Dennie Parker, Jennifer Richards, Nina Ritchie, Kristen C. Roessler, Sharon Saydah, Kim Taylor, Puthiery Va, Dan VanDeRiet, Del Yazzie, Laura L. Hammitt, Catherine G. Sutcliffe

**Affiliations:** 1 Department of International Health, Johns Hopkins Bloomberg School of Public Health, Baltimore, Maryland, United States of America; 2 Tuba City Regional Health Care Corporation, Tuba City, Arizona, United States of America; 3 Indian Health Service, Rockville, Maryland, United States of America; 4 Whiteriver Service Unit, Phoenix Area, Indian Health Service, Whiteriver, Arizona, United States of America; 5 Navajo Area Indian Health Service, St. Michaels, Arizona, United States of America; 6 Coronovirus and Other Respiratory Viruses Division, Centers for Disease Control and Prevention, Atlanta, GeorgiaUnited States of America; 7 Division of Intramural Research, National Institute of Allergy and Infectious Diseases, National Institutes of Health, Bethesda, Maryland, United States of America; 8 Department of Pathology, Division of Medical Microbiology, Johns Hopkins School of Medicine, Baltimore, Maryland, United States of America; 9 Chinle Service Unit, Navajo Area, Indian Health Service, Chinle, Arizona, United States of America; 10 Navajo Epidemiology Center, Window Rock, Arizona, United States of America; 11 Department of Epidemiology, Johns Hopkins Bloomberg School of Public Health, Baltimore, Maryland, United States of America; PLOS: Public Library of Science, UNITED STATES OF AMERICA

## Abstract

Long-term effects of COVID-19 on multiple organ systems have been reported. Indigenous persons experienced disproportionate morbidity and mortality from COVID-19; however, Post-COVID-19 Conditions (PCC) have not been well described in this population. We conducted a longitudinal cohort study among Indigenous persons living in the Navajo Nation or White Mountain Apache Tribal lands in the Southwest United States who tested positive for SARS-CoV-2 between February 1, 2021 and August 31, 2022. Participants were enrolled during their acute illness and followed for three months. PCC was defined as the presence of any self-reported symptom and/or any sequelae or new condition recorded in the electronic health record at the 3-month visit. Risk factors for PCC were evaluated using Poisson regression with robust standard errors. The analysis included 258 adults and 84 children. Most participants (98.4% of adults, 90.5% of children) experienced a mild, symptomatic acute illness. Over half of adults (57.8%) and a third (39.3%) of children experienced six or more symptoms during the acute illness. Three months post-acute COVID-19, 39.8% of adults and 15.9% of children had symptoms consistent with PCC. Commonly reported symptoms were fatigue/tiredness, cough, headache, runny nose, and myalgia. Among adults enrolled during Omicron predominance, older age and hospitalization for COVID-19 were significantly associated with an increased risk of PCC, and COVID-19 vaccination was significantly associated with a decreased risk of PCC in univariable analysis. In a multivariable analysis, COVID-19 vaccination (risk ratio: 0.56; 95% confidence interval: 0.34, 0.90) remained significantly associated with a decreased risk of PCC. In this cohort of Indigenous persons in the Southwest US, PCC at three months post-acute COVID-19 illness were common, including among individuals with mild acute illness. While the absence of a control group is a limitation, these findings highlight the potential ongoing healthcare needs related to PCC in Indigenous populations.

## Introduction

Throughout the COVID-19 pandemic, underserved populations in the United States, including Indigenous persons, have experienced disproportionate burden from COVID-19. When adjusted for age, persons classified as American Indian and Alaska Native (AI/AN) were 1.6 times more likely to become infected, 2.4 times more likely to be hospitalized, and 2.0 times more likely to die because of COVID-19 than non-Hispanic White Americans, even among individuals with lower levels of co-morbidities [1,2].

Long-term effects on multiple organ systems following acute COVID-19 have been described [3–5], although these effects and potential risk factors are not as well understood as those associated with acute illness. Post COVID-19 Conditions (PCC), Long COVID, long haul COVID, post-acute COVID-19 syndrome, and post-acute sequelae of COVID-19 (PASC) have all been used to describe these long-term effects, or generally, the occurrence of signs and/or symptoms that develop or persist 4 weeks or longer after acute COVID-19 has resolved [6,7]. Most common symptoms reported by those experiencing PCC include new or persistent respiratory symptoms, fatigue (with or without bodily pain), and/or cognitive impairment (“brain fog”) [6,8–10]. PCC has been shown to interfere with daily function and productivity, and can lead to increased healthcare visits and loss of income, among other impacts on quality of life [11–13]. Female sex, older age, presence of underlying medical conditions (e.g., obesity), and severity of acute COVID-19 illness are consistently reported as risk factors for PCC [14,15]. Considering the high prevalence of chronic comorbid conditions and limited access to healthcare, especially subspeciality services, in rural Indigenous communities, managing PCC could prove challenging for Indigenous persons and their healthcare providers. Information on the prevalence of and risk factors for PCC among Indigenous persons is limited, but may inform culturally-grounded and relevant PCC management [16].

This study was conducted to characterize acute COVID-19 symptoms and occurrence of short-term PCC, and risk factors for PCC in Indigenous persons, who have been disproportionately burdened by COVID-19 morbidity and mortality.

## Methods

### Ethics and consent

This study was reviewed and approved by the Johns Hopkins Bloomberg School of Public Health Institutional Review Board (IRB 9605/MOD00000534) and the ethics review board for each participating tribe (Navajo Nation Human Research Review Board [NNR-19.350] and Phoenix Area Indian Health Service [IHS] IRB [PXR 19.08]). The Centers for Disease Control and Prevention (CDC) IRB relied on the Johns Hopkins IRB [17]. This study followed the Strengthening the Reporting of Observational Studies in Epidemiology (STROBE) reporting guidelines [18]. Adult participants provided written informed consent for participation in the study. Written parental permission was obtained for participants aged less than 18 years; in addition, written or oral assent was obtained for minor participants aged 7 to 17 years.

### Inclusivity in global research

Additional information regarding the ethical, cultural, and scientific considerations specific to inclusivity in global research is included in the Supporting Information (S1 Checklist)

### Study design and population

This prospective cohort study of persons testing positive for SARS-CoV-2 was conducted among Indigenous persons of all ages living in the rural communities of Chinle, AZ and Tuba City, AZ (both in the Navajo Nation), and in Whiteriver, AZ in the White Mountain Apache Tribal lands. Healthcare is provided to tribal members through IHS hospitals, tribal healthcare facilities, and private healthcare facilities. Potential participants were identified through active facility-based surveillance that was ongoing in these communities [19,20]. Participants were recruited from inpatient facilities, outpatient facilities (Emergency Departments or outpatient clinics), and SARS-CoV-2 testing clinics. At inpatient facilities, staff conducted screening six days per week to recruit patients hospitalized with COVID-19. At outpatient facilities, staff recruited symptomatic COVID-19 patients two to four days per week. At SARS-CoV-2 testing clinics, staff recruited asymptomatic or symptomatic patients who tested positive for SARS-CoV-2 two to four days per week. After enrollment in the study, participants had a baseline visit (i.e., the acute illness and enrollment visit) and follow-up visits 1 and 3 months later.

The study was designed with a target sample size of 320-380 participants. Given the paucity of information on PCC early in the pandemic, this sample size was determined to be sufficient to estimate the prevalence of PCC (+/- 5%) and identify the risk factors of greatest interest (risk ratio>2.5) for sequelae.

This analysis was adapted from research findings originally published in a dissertation [21].

### Data collection

Enrollment began after local permissions were obtained (February 9, 2021 in Chinle, March 15, 2021 in Whiteriver, and May 17, 2021 in Tuba City); all sites concluded enrollment on August 31, 2022. At the baseline visit, information on participants’ sociodemographic and household characteristics, self-reported symptoms, and self-reported COVID-19 vaccination status was collected by interviewing the adult (≥18 years) participant, the parent/guardian of child (<7 years) participants, and either the parent/guardian or participant for youth/adolescent (7-17 years) participants. Mid-turbinate nasal swab specimens were collected by research personnel at this visit; if a research specimen could not be collected, a residual clinical nasal swab specimen collected by healthcare staff was used instead if available. Mid-turbinate nasal swabs were placed in viral transport media, aliquoted, and stored at -80°C until testing, which occurred 65 to 214 days following collection. Specimens were sent to Johns Hopkins University for viral genomic sequencing to determine SARS-CoV-2 lineage. Specimens were extracted using the Chemagic 360 system (Perkin Elmer) using methods and procedures previously described [22]. A blood sample was also collected via venipuncture for SARS-CoV-2 antibody testing. Specimens were processed and stored as serum at -80°C until testing, which occurred 117 to 341 days following collection. Specimens were sent to Johns Hopkins University for serology testing for anti-nucleocapsid, anti-spike, and anti-RBD IgG antibodies using the Meso Scale Discovery (MSD) V-PLEX SARS-CoV-2 Panel 2 (IgG) Kit^TM^ following the manufacturer’s protocol [23]*.* The assay has a reported sensitivity/specificity for anti-nucleocapsid and spike IgG of 93.8%/100.0% and 98.3%/99.5%, respectively [23].

One month after the baseline visit, electronic health records (EHR) were reviewed to collect information regarding the participant’s health status prior to enrollment, including underlying medical conditions, previous testing for SARS-CoV-2 (including date, test type, and results), and COVID-19 vaccines received (state immunization information systems (IIS) were also reviewed). Information was also collected on the clinical course, COVID-19 treatment received, and outcome of the acute illness.

At the 3-month follow-up visit, interviews were conducted to assess recovery and lasting effects using a standardized questionnaire. EHRs were also reviewed to collect information about signs and symptoms post-acute infection, additional COVID-19 vaccination(s) received, healthcare visits associated with COVID-19, and new medical conditions.

### Definitions

This study utilized self-reported data regarding symptoms after the acute illness, and data ascertained via chart review regarding signs and symptoms and diagnosis of new medical conditions since the acute illness to evaluate PCC (see S1 Table). The list of signs and symptoms and diagnoses included in the questionnaires and chart review were developed based on the CDC Person Under Investigation symptom list, CDC Symptom Inventory [24], and information available at the time the study started [5]. In this analysis, PCC was defined as the presence of any self-reported symptom (new or persistent) that the participant thought to be related to their acute COVID-19 illness at the 3-month visit, any signs and symptoms of COVID-19 recorded in the EHR after the acute illness and up to the 3-month visit, and/or new report of one of the following medical conditions in the EHR after the acute illness and up to the 3-month visit: autoimmune condition, cardiovascular disease, cardiac dysrhythmia, heart failure, acute myocardial infarction, acute pulmonary embolism, asthma, renal failure (dialysis), chronic kidney disease, coagulation and hemorrhagic event, thromboembolic event, cerebrovascular event, gastrointestinal and esophageal event, neurologic conditions, sleeping disorders, mental health conditions, substance-related disorders, muscle disorder, and/or diabetes types 1 and 2 [5].

Discrepancies between EHR-documented and self-reported COVID-19 vaccination status were investigated, including re-contacting participants if appropriate; dates from EHR/IIS were used unless these were missing. Participants were considered to have completed their primary series if they had received two doses of an approved mRNA COVID-19 vaccine or one dose of an approved non-mRNA vaccine ≥14 days prior to illness onset (with or without any booster doses).

Participants were considered to have had a previous SARS-CoV-2 infection if they reported having had SARS-CoV-2 prior to the baseline visit, if a prior SARS-CoV-2 infection was noted in the EHR, or if they had serologic evidence of prior SARS-CoV-2 infection. Serologic evidence of prior SARS-CoV-2 infection was based on nucleocapsid antibody positivity among participants with a blood sample taken within one week of illness onset only, to ensure presence of antibodies were not the result of the current infection.

Variant predominant periods were defined according to when a particular SARS-CoV-2 variant accounted for >50% of sequenced samples, using national trends [25] and corroborated by viral genomes sequenced as part of the current study, as not all study specimens were able to be sequenced.

### Statistical analysis

All analyses were performed in Stata SE version 17 (StataCorp LLC, College Station, Texas).

### Characterizing acute COVID-19 illness

The prevalence of individual symptoms and, among those with a medical encounter during their acute illness, clinical metrics (days between symptom onset and presentation at health care facility, receipt of COVID-19 treatments, receipt of supplemental oxygen, length of hospital stay, intensive care unit [ICU] admission, and death) were summarized. Symptoms were included if documented by participant self-report or ascertained via chart review. Continuous variables were expressed as medians and interquartile ranges as not all variables were normally distributed. Categorical variables were summarized as counts and percentages. Participants missing information for a given variable were excluded from the summary estimates for that variable. All analyses were stratified by age group (“adults” being 18 years or older and “children” being younger than 18 years old). Analyses of symptoms and outcomes of the acute illness were additionally stratified by level of care provided (outpatient vs inpatient). For children, as the number of participants hospitalized was small (n<10), only results for all participants and outpatients are reported.

### Characterizing the occurrence and risk factors for PCC

The proportion of participants with self-reported symptoms, documented signs and symptoms, and/or new conditions recorded at three months was calculated to characterize PCC. Risk factors for PCC were evaluated separately for adults and children. Covariates assessed included age (18-49, 50-64, or ≥65 years), sex (male or female), enrollment site, level of education (some high school or less, high school diploma or GED, some college or AA, completed degree including graduate), access to running water in home (yes or no), wood used to heat home (yes or no), smoking status (current or former smoker or never smoker), underlying medical conditions, vaccination status (unvaccinated or primary series +/- booster dose(s)), number of days between symptom onset and presentation for care (0-5 or ≥6 days), symptoms present at baseline (yes or no), serologic evidence of prior infection at baseline (yes or no), self-reported SARS-CoV-2 infection (yes or no), and severity of acute COVID-19 illness (inpatient or outpatient). Presence of underlying conditions was assessed overall (i.e., any underlying medical condition), by individual condition, and using a composite variable of select conditions. The proportion with PCC among those with and without a given characteristic was compared using chi-square test (or Fischer’s exact test when appropriate). Given the small sample size among children throughout the study period and the small sample size of adults prior to Omicron predominance, the final risk factor analysis was restricted to adults enrolled during Omicron predominance. Risk ratios (RR) of PCC and 95% confidence intervals (CIs) were estimated using Poisson regression with robust standard errors [26]. CIs that did not include 1.00 were considered statistically significant. Missing data were handled through listwise deletion given the small (<10%) amount of missing data for most covariates. After checking for collinearity, covariates that were statistically significant in bivariable analysis (medical presentation at acute illness, COVID-19 vaccination status), or were not statistically significant but were pre-established risk factors (age, sex, underlying medical condition[s]) [14,15], were included in the final multivariable model.

A sensitivity analysis was conducted for the risk factor analysis to assess the impact of the PCC definition on the covariates identified. The risk factor analysis was repeated, restricting the PCC definition to self-reported symptoms thought to be related to the acute illness (i.e., excluding signs and symptoms and new diagnoses recorded in the EHR).

## Results

### Characteristics of study participants

Three hundred and fifty-one patients with COVID-19 were enrolled; 342 participants completed the baseline visit and chart review and were included in the evaluation of acute illness (S1 Fig).

The majority of participants were female (66.1%, 226/342) and enrolled at the Chinle site (60.8%, 208/342); 24.6% were <18 years old (84/342), 42.7% were ages 18–49 years (146/342), and 32.7% were ≥50 years (112/342) (Table 1). At baseline, the most common underlying medical conditions among adults were diabetes (type 1 or 2, 32.9%, 85/258), obesity (32.6%, 84/258), hypertension (27.1%, 70/258), and mental health conditions (20.9%, 54/258). Among children, obesity (19.0%, 16/84) and asthma (14.3%, 12/84) were the most common underlying medical conditions. Most (82.9%, 214/258) adults and less than half (42.9%, 36/84) of children had completed the primary series of a COVID-19 vaccine prior to their acute illness. At the time of the acute illness, 51.1% (96/188) of adults and 43.2% (16/37) of children had evidence of prior infection, as evidenced by nucleocapsid antibody positivity or self-reported prior infection (Table 1). Almost all (95.2%, 119/125) adults and 66.7% (20/30) of children had serological evidence of spike glycoprotein antibodies (S2 Fig), indicating either prior COVID-19 vaccination and/or prior SARS-CoV-2 infection. Specimens from 168 individuals were successfully sequenced. Of these, 30.4% (51/168) were clade 21A, 21I, or 21J (Delta) and 64.3% (108/168) were clade 21K–M or 22A–C (Omicron or an Omicron sub-lineage) (S3 Fig). Among sequenced specimens, Delta was the predominant variant between July and December 2021 and Omicron and sub-lineages were predominant starting in January 2022.

**Table 1:** Sociodemographic characteristics of participants at baseline, overall and by age group.

	Total (N=342)	Adults (≥18 years)			Children (<18 years)	
		Total (N=258)	Outpatient (N=220)	Inpatient (N=38)	Total (N=84)^g^	Outpatient (N=76)
	n (%)	n (%)	n (%)	n (%)	n (%)	n (%)
**Median age in years (IQR)**	40.2 (18.7-55.4)	46.3 (35.2-58.5)	46.1 (34.6-58.1)	53.9 (42.9, 59.5)	10.6 (5.4-14.3)	11.7 (6.6, 14.5)
**Age group (in years)**						
<5	20 (5.8)	NA	NA	NA	20 (23.8)	14 (18.4)
5 to 17	64 (18.7)	NA	NA	NA	64 (76.2)	62 (81.6)
18 to 49	146 (42.7)	146 (56.6)	130 (59.1)	16 (42.1)	NA	NA
50 to 64	76 (22.2)	76 (29.5)	61 (27.3)	15 (39.5)	NA	NA
≥65	36 (10.5)	36 (14.0)	29 (13.2)	7 (18.4)	NA	NA
**Female sex**	226 (66.1)	186 (72.1)	160 (72.7)	26 (68.4)	40 (47.6)	36 (47.4)
**Enrollment site (service unit)**						
Chinle	208 (60.8)	154 (59.7)	128 (58.5)	26 (68.4)	54 (64.3)	46 (60.5)
Tuba City	77 (22.5)	53 (20.5)	47 (21.5)	6 (15.8)	24 (28.6)	24 (31.6)
Whiteriver	56 (16.4)	50 (19.4)	44 (20.1)	6 (15.8)	6 (7.1)	6 (7.9)
**Running water in home** ^a^	272 (79.5)	210 (81.4)	186 (72.1)	24 (9.3)	62 (73.8)	57 (75.0)
**Wood used to heat home**	221 (64.6)	165 (64.0)	139 (63.2)	26 (68.4)	56 (66.7)	51 (67.1)
**Education level** ^a^ ^,^ ^b^						
Some high school or less	38 (11.1)	24 (9.3)	19 (8.6)	5 (13.2)	14 (16.7)	13 (17.1)
High school diploma or GED	110 (32.2)	83 (32.2)	72 (32.7)	11 (28.9)	27 (32.1)	24 (31.6)
Some college or AA	131 (38.3)	108 (41.9)	95 (43.2)	13 (34.2)	23 (27.4)	21 (27.6)
Completed degree, including graduate	35 (10.2)	26 (10.1)	26 (11.8)	0 (0.0)	9 (10.7)	9 (11.8)
**Medical condition or COVID-19 risk factor present**						
Alcohol and/or substance abuse	24 (7.0)	24 (9.3)	17 (7.7)	7 (18.4)	0 (0.0)	0 (0.0)
Asthma	49 (14.3)	37 (14.3)	30 (13.6)	7 (18.4)	12 (14.3)	11 (14.5)
Chronic lung disease	7 (2.0)	7 (2.7)	7 (3.2)	0 (0.0)	0 (0.0)	0 (0.0)
Current or former smoker	47 (18.8)	47 (18.8)	36 (16.7)	11 (31.4)	NA	NA
Chronic kidney disease	7 (2.0)	6 (2.3)	4 (1.8)	2 (5.3)	1 (1.2)	1 (1.3)
Chronic liver disease	11 (3.2)	11 (4.3)	10 (4.5)	1 (2.6)	0 (0.0)	0 (0.0)
Diabetes (type 1 or 2)	85 (24.8)	85 (32.9)	60 (27.3)	25 (65.8)	0 (0.0)	0 (0.0)
Heart condition, excluding hypertension	24 (7.0)	23 (8.9)	14 (6.4)	9 (23.7)	1 (1.2)	1 (1.3)
Hypertension	72 (21.0)	70 (27.1)	53 (24.1)	17 (44.7)	2 (2.4)	2 (2.6)
Immunocompromised	4 (1.2)	4 (1.6)	2 (0.9)	2 (5.3)	0 (0.0)	0 (0.0)
Mental health condition	55 (16.0)	54 (20.9)	44 (20.0)	10 (26.3)	1 (1.2)	1 (1.3)
Obesity	100 (29.2)	84 (32.6)	68 (30.9)	16 (42.1)	16 (19.0)	14 (18.4)
Supplemental oxygen use at home	9 (2.6)	9 (3.5)	1 (0.5)	8 (21.1)	0 (0.0)	0 (0.0)
**COVID-19 vaccination status at time of acute illness** ^c^						
Unvaccinated	92 (26.9)	44 (17.1)	30 (13.6)	14 (36.8)	48 (57.1)	40 (52.6)
Completed primary series only	184 (53.8)	153 (59.3)	137 (62.3)	16 (42.1)	31 (36.9)	31 (40.8)
Completed primary series + ≥1 booster dose	66 (19.3)	61 (23.6)	53 (24.1)	8 (21.1)	5 (6.0)	5 (6.6)
**Medical presentation for acute illness**						
Outpatient^d^	296 (86.5)	220 (85.3)	NA	NA	76 (90.5)	NA
Inpatient	46 (13.5)	38 (14.7)	NA	NA	8 (9.5)	NA
**Symptomatic acute illness**	330 (96.5)	254 (98.4)	216 (98.2)	38 (100)	76 (90.5)	68 (89.5)
**Self-reported or serologic evidence of prior SARS-CoV-2 infection** ^e^						
No	113 (50.2)	92 (48.9)	9 (34.6)	83 (51.2)	21 (56.8)	21 (58.3)
Yes	112 (49.8)	96 (51.1)	17 (65.4)	79 (48.8)	16 (43.2)	15 (41.7)
**Variant predominance for acute illness** ^a^ ^,^ ^f^						
Pre-Omicron	126 (36.8)	94 (36.4)	75 (34.1)	19 (50.0)	32 (38.1)	30 (39.5)
During Omicron and sub-variants	216 (63.2)	164 (63.6)	154 (65.9)	19 (50.0)	52 (61.9)	56 (60.5)

AA, Associate of Arts degree; GED, General Educational Development; IQR, interquartile range.

^a^28 (8.2%) participants missing education level; 7 (2.1%) missing running water in home.

^b^Education status of participant; if participant <18 years old, education status of mother.

^c^Completed primary series only = Received two doses of an approved mRNA COVID-19 vaccine primary series or one dose of an approved non-mRNA vaccine ≥14 days prior to illness onset. Completed primary series +≥1 booster dose = Completed primary COVID-19 vaccine series and also received ≥1 booster dose.

^d^Outpatient = participants enrolled at outpatient clinics, Emergency Departments, or SARS-CoV-2 testing clinics.

^e^Serologic evidence of prior infection determined by blood specimen positive for nucleocapsid IgG antibody. Only blood specimens collected within 1 week of illness onset were included (total n=155; adults n=125; children n=30). 70 (27.1%) adults and 47 (55.9%) children were missing self-reported prior infection and/or serologic evidence.

^f^Variant predominance was defined as the period during which Omicron was detected in >50% of sequenced cases using national trends [25], and corroborated by viral genomes sequenced as part of the current study. Omicron was the predominant variant from December 25, 2021 onwards.

^g^Data on inpatient children not presented because of sparse data (N<10)

### Characterizing acute COVID-19 illness

Among adult participants, 98.4% (254/258) had symptomatic acute illness (Table 1). Among adults who experienced symptoms, the most common were cough (85.4%, 217/254), headache (69.7%, 177/254), runny nose (57.5%, 146/254), myalgia (49.2%, 125/254), and sore or itchy throat (48.8%, 124/254); 58.7% (149/254) experienced six or more symptoms (Table 2). Among child participants, 90.5% (76/84) had symptomatic acute illness (Table 1). Among children who experienced symptoms, the most common included cough (75.0%, 57/76), runny nose (69.7%, 53/76), sore or itchy throat (51.3%, 39/76), and fever (47.4%, 36/76); 43.4% experienced six or more symptoms (33/76) (Table 2). Symptoms ascertained by self-report and chart review are presented separately for adults and children in S2 and S3 Tables, respectively. Generally, proportions of symptom categories (e.g., respiratory, gastrointestinal) ascertained via self-report were similar to those ascertained by EHR, with the exception of neurologic and systemic symptoms where symptoms were more common from self-report than EHR. Greater discordance was observed for some individual symptoms between self-report and EHR (e.g., chills, fatigue).

**Table 2:** Symptoms self-reported and/or documented in the electronic health record during the acute COVID-19 illness, by hospitalization status and age group.

	Adults (≥18 years)^a^			Children (<18 years)^a^	
	Total (N=254)	Inpatient (n=38)	Outpatient (n=216)	Total (N=76)^b^	Outpatient (n=68)
	n (%)	n (%)	n (%)	n (%)	n (%)
**Systemic**	204 (80.3)	34 (89.5)	170 (78.7)	53 (69.7)	46 (67.6)
Chills/rigors	109 (42.9)	20 (52.6)	89 (41.2)	17 (22.4)	17 (25.0)
Difficulty sleeping	1 (0.4)	0 (0.0)	1 (0.5)	0 (0.0)	0 (0.0)
Fatigue and/or tiredness^c^	101 (39.8)	18 (47.4)	83 (38.4)	11 (14.5)	8 (11.8)
Fever	113 (44.5)	25 (65.8)	88 (40.7)	36 (47.4)	30 (44.1)
Malaise	4 (1.6)	0 (0.0)	4 (1.9)	1 (1.3)	1 (1.5)
Sepsis or shock	0 (0.0)	0 (0.0)	0 (0.0)	0 (0.0)	0 (0.0)
Weak/dizzy	84 (33.1)	17 (44.7)	67 (31.0)	13 (17.1)	12 (17.6)
**Respiratory**	190 (74.8)	28 (73.7)	162 (75.0)	58 (76.3)	51 (75.0)
Acute respiratory distress	0 (0.0)	0 (0.0)	0 (0.0)	0 (0.0)	0 (0.0)
Apnea	0 (0.0)	0 (0.0)	0 (0.0)	0 (0.0)	0 (0.0)
Chest pain and/or tightness	49 (19.3)	15 (39.5)	34 (15.7)	7 (9.2)	6 (8.8)
Cough	217 (85.4)	37 (97.4)	180 (83.3)	57 (75.0)	51 (75.0)
Decreased breathing sounds	6 (2.4)	5 (13.2)	1 (0.5)	0 (0.0)	0 (0.0)
Pain when coughing	1 (0.4)	0 (0.0)	1 (0.5)	0 (0.0)	0 (0.0)
Pneumonia	8 (3.1)	6 (15.8)	2 (0.9)	0 (0.0)	0 (0.0)
Rales	9 (3.5)	9 (23.7)	0 (0.0)	0 (0.0)	0 (0.0)
Respiratory distress	2 (0.8)	2 (5.3)	0 (0.0)	1 (1.3)	0 (0.0)
Retractions	0 (0.0)	0 (0.0)	0 (0.0)	4 (5.3)	0 (0.0)
Shortness of breath	92 (36.2)	29 (76.3)	63 (29.2)	12 (15.8)	7 (10.3)
Sputum production	75 (29.5)	18 (47.4)	57 (26.4)	14 (18.4)	11 (16.2)
Stridor	1 (0.4)	1 (2.6)	0 (0.0)	1 (1.3)	0 (0.0)
Tachypnea	4 (1.6)	4 (10.5)	0 (0.0)	2 (2.6)	0 (0.0)
Wheeze	57 (22.4)	18 (47.4)	39 (18.1)	8 (10.5)	6 (8.8)
**Head, ear, nose, throat**	222 (87.4)	26 (68.4)	196 (90.7)	63 (82.9)	57 (83.8)
Congestion	105 (41.3)	10 (26.3)	95 (44.0)	15 (19.7)	13 (25.0)
Conjunctivitis	25 (9.8)	6 (15.8)	19 (8.8)	7 (9.2)	4 (5.9)
Ear pain	10 (3.9)	0 (0.0)	10 (4.6)	0 (0.0)	0 (0.0)
Headache	177 (69.7)	22 (57.9)	155 (71.8)	32 (42.1)	31 (45.6)
Runny nose	146 (57.5)	17 (44.7)	129 (59.7)	53 (69.7)	47 (69.1)
Sinus pain	4 (1.6)	0 (0.0)	4 (1.9)	0 (0.0)	0 (0.0)
Sneezing	1 (0.4)	0 (0.0)	1 (0.5)	0 (0.0)	0 (0.0)
Sore/itchy throat	124 (48.8)	12 (31.6)	112 (51.9)	39 (51.3)	38 (55.9)
**Cognitive**					
Confusion	26 (10.2)	8 (21.1)	18 (8.3)	2 (2.6)	2 (2.9)
**Neurologic**	76 (29.9)	13 (34.2)	63 (29.2)	19 (25.0)	18 (26.5)
Loss of taste or smell	75 (29.5)	12 (31.6)	63 (29.2)	18 (23.7)	17 (25.0)
Seizure	1 (0.4)	1 (2.6)	0 (0.0)	1 (0.5)	1 (1.5)
**Circulatory**	9 (3.5)	8 (21.1)	1 (0.5)	0 (0.0)	0 (0.0)
Cyanosis	0 (0.0)	0 (0.0)	0 (0.0)	0 (0.0)	0 (0.0)
Hypoxemia	9 (3.5)	8 (21.1)	1 (0.5)	0 (0.0)	0 (0.0)
**Cardiac**					
Tachycardic	5 (2.0)	0 (0.0)	5 (2.3)	1 (1.3)	1 (1.5)
**Gastrointestinal**	100 (39.4)	18 (47.4)	82 (38.0)	31 (40.8)	26 (38.2)
Abdominal/stomach pain	19 (7.5)	6 (15.8)	13 (6.0)	1 (1.3)	0 (0.0)
Diarrhea	47 (18.5)	14 (36.8)	33 (15.3)	12 (15.8)	11 (16.2)
Loss of appetite	58 (22.8)	12 (31.6)	46 (21.3)	12 (15.8)	9 (13.2)
Nausea	60 (23.6)	14 (36.8)	46 (21.3)	12 (15.8)	11 (16.2)
Transaminitis	1 (0.4)	0 (0.0)	1 (0.5)	0 (0.0)	0 (0.0)
Vomiting	25 (9.8)	10 (26.3)	15 (6.9)	20 (26.3)	13 (19.1)
**Musculoskeletal**	125 (49.2)	20 (52.6)	105 (48.6)	32 (42.1)	31 (45.6)
Muscle/body aches	125 (49.2)	20 (52.6)	105 (48.6)	32 (42.1)	31 (45.6)
Red or bruised toes	4 (1.6)	2 (5.7)	2 (0.9)	0 (0.0)	0 (0.0)
Rash	1 (0.4)	0 (0.0)	1 (0.5)	1 (1.3)	1 (1.5)
**Other** ^d^	18 (7.1)	7 (18.4)	11 (5.1)	2 (2.6)	2 (2.9)
**≥6 symptoms**	149 (58.7)	22 (57.9)	127 (58.8)	33 (43.4)	29 (42.6)

Note: Totals for all individual symptoms (e.g., fever, malaise) per category (e.g., systemic) may not equal category total because categories were defined as at least one of all individual symptoms (i.e., a participant with fever, chills/rigors, and malaise would contribute the same to the systemic total as a participant with only fever).

^a^Table restricted to symptomatic participants; four asymptomatic outpatient adults and eight asymptomatic outpatient children not included.

^b^Data on inpatient children not presented because of sparse data (N<10).

^c^Fatigue and/or tiredness includes “abnormally sleepy,” “fatigue,” “lethargy,” and “tiredness” self-reported and/or documented in the electronic health record.

^d^Other included the following: one episode of vomiting blood; leg swelling; red bump on left cheek; blurred vision, watery eyes; intermittent burning sensation in hands and feet; sweats; myalgia, possibly due to arthritis and not COVID-19; feels dehydrated; coughing up blood, bloody nose, muscle cramps; mild metabolic acidosis; anxiety attack; shoulder pain; tooth pain; thirsty; “barely audible” expiration diffusion; allergies; thirsty; light sensitivity, constipation.

To assess whether symptoms varied by SARS-CoV-2 variant, symptoms experienced during the acute illness were compared between the pre-Omicron and Omicron periods (S4 Table). The analysis was restricted to outpatient participants due to the limited number of inpatients in each period. Significantly more adults reported loss of taste or smell, pneumonia, and tachycardia prior to Omicron predominance, and significantly more reported muscle/body aches and sore or itchy throat during Omicron predominance. Among children, significantly more reported chills or rigors, fever, weakness or dizziness, cough, sputum production, and sore or itchy throat during Omicron predominance (S4 Table).

More than half (58.9%, 152/258) of adult participants received at least one treatment for their acute COVID-19 illness; few children (4.8%, 4/84) received treatment (Table 3). Adult inpatients were commonly treated with steroids and antivirals (i.e., remdesivir), while monoclonal antibodies and antivirals (i.e., nirmatrelvir-ritonavir) were commonly used to treat outpatients prior to and during Omicron predominance, respectively (results not shown).

**Table 3:** Clinical outcomes and COVID-19 treatment during acute illness, by hospitalization status and age group.

	Adults (≥18 years)			Children (<18 years)	
	Total (N=258)	Inpatient (n=38)	Outpatient^a^ (n=220)	Total (N=84)^b^	Outpatient^a^ (n=76)
	n (%)	n (%)	n (%)	n (%)	n (%)
**Days between illness onset and presenting for care**					
0 to 5	205 (79.5)	25 (65.8)	180 (81.8)	78 (92.9)	71 (93.4)
6 to 10	33 (12.8)	11 (29.0)	22 (10.0)	3 (3.6)	2 (2.6)
≥11	20 (7.8)	2 (5.3)	18 (8.2)	3 (3.6)	3 (4.0)
**Given ≥1 treatment for COVID-19** ^c^	152 (58.9)	33 (86.8)	119 (54.1)	4 (4.8)	0 (0.0)
Convalescent plasma	0 (0.0)	0 (0.0)	0 (0.0)	0 (0.0)	0 (0.0)
Monoclonal antibodies	91 (35.3)	7 (18.4)	84 (38.2)	0 (0.0)	0 (0.0)
Steroids	26 (10.1)	25 (65.8)	2 (0.9)	4 (4.8)	0 (0.0)
Antivirals^d^	64 (24.8)	30 (78.9)	34 (15.5)	2 (2.4)	0 (0.0)
Remdesivir	30 (11.6)	29 (76.3)	1 (0.5)	2 (2.4)	0 (0.0)
Nirmatrelvir-ritonavir	36 (14.0)	3 (7.9)	33 (15.0)	0 (0.0)	0 (0.0)
Other treatment for COVID-19^e^	1 (0.4)	1 (2.6)	0 (0.0)	0 (0.0)	0 (0.0)
**Received supplemental oxygen**	30 (11.6)	28 (73.7)	2 (0.9)	4 (4.8)	0 (0.0)
**Among hospitalized**					
Median length of stay in days (IQR)	NA	3.0 (2.0-5.0)	NA	NA	NA
ICU admission	NA	3 (7.9)	NA	NA	NA
Severe^f^	NA	3 (7.9)	NA	NA	NA
**Died as a** **result of acute illness**^g^	2 (0.8)	2 (5.3)	NA	0 (0.0)	0 (0.0)

ICU, intensive care unit; IQR, interquartile range; NA, not applicable.

^a^Outpatient = participants enrolled at outpatient clinics, Emergency Departments, or SARS-CoV-2 testing clinics.

^b^Data on inpatient children not presented because of sparse data (N<10).

^c^Treatment for COVID-19 was assessed using EHR. One adult (0.4%) and six children (7.1%) do not have information on treatment received for acute COVID-19.

^d^No participants were documented as receiving other antivirals, including Amantadine, Baloxavir marboxil, Oseltamivir, Peramivir, Ribavirin, or Zanamivir.

^e^One adult received Famotidine during the course of their COVID-19 acute illness.

^f^Severe = Severe COVID-19-associated hospitalization was defined among patients with COVID-19-associated hospitalization according to the World Health Organization’s definition and included at least one of the following clinical outcomes occurring during the course of illness: use of supplemental oxygen by non-invasive ventilation or high-flow nasal cannula; intubation or mechanical ventilation; use of vasopressors; dialysis; extracorporeal membrane oxygenation (ECMO); or death [27].

^g^One additional adult died 1 month and 3 days after hospitalization, but cause of death was not available in the medical records.

Among 38 hospitalized adults, three (7.9%) were admitted to an ICU (Table 3). Most hospitalized adults received supplemental oxygen during their admission (73.7%, 28/38). The median length of hospital stay was 3 (Interquartile range [IQR]: 2–5) days. Two (5.3%, 2/38) hospitalized adults died because of their acute COVID-19 illness.

### Characterizing occurrence and risk factors for PCC

**Fig 1:**
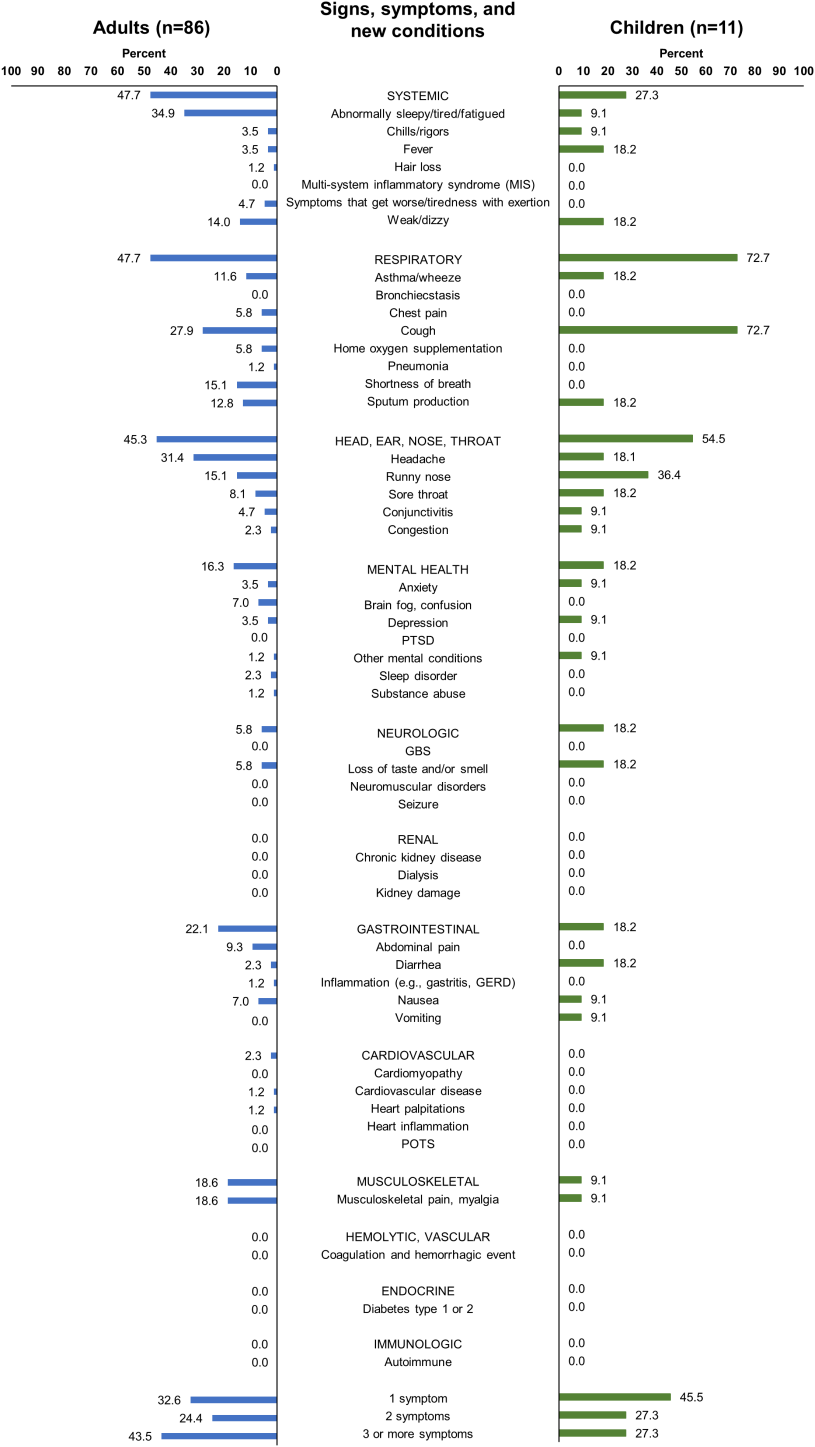
Manifestations of Post-COVID-19 Conditions (PCC) three months post-acute illness, among those with PCC, by age group^a^. GBS, Guillain-Barré syndrome; GERD, Gastroesophageal reflux disease; POTS, Postural tachycardia syndrome; PTSD, Post-traumatic stress disorder. ^a^PCC determined via self-reported symptoms during the 3-month visit interview, signs and symptoms documented in the electronic health record three months post-acute illness, and/or new conditions documented in the electronic health record three months post-acute illness.

Two hundred and sixteen adults had complete information from their 3-month visit and were included in the PCC assessment and risk factor analysis (Fig 1). Of those, 86 (39.8%, 86/216) met criteria for PCC. Seventy-two (37.1%, 72/194) presented as outpatients during acute illness and 14 (63.6%, 14/22) presented as inpatients; 26 (36.6%, 26/71) adults with PCC presented with acute illness prior to Omicron predominance and 60 (41.4%, 60/145) presented with acute illness during Omicron predominance (Table 4, S5 Table). Only four of the 216 adults included in the analysis had asymptomatic acute illness: none of the four developed symptoms consistent with PCC, compared to 40.6% (86/212) of adults who were symptomatic during their acute illness (p-value=0.10). Common manifestations of PCC among adults were fatigue and/or tiredness (34.9%, 30/86), headache (31.4%, 27/86), cough (27.9%, 24/86), myalgia (18.6%, 16/86), and shortness of breath (15.1%, 13/86); 43.0% (37/86) of adults with PCC experienced at least three symptoms (Fig 1). Symptoms via self-report, signs and symptoms from the EHR, and new conditions are reported separately in S6–S8 Tables. They show that adults had more signs and symptoms by self-report than EHR, and few new conditions noted in EHR 3 months post infection.

**Table 4:** Sociodemographic and clinical characteristics associated with PCC at three months post-acute illness among adults enrolled during Omicron predominance.

	Total (N=145)	With PCC (n=60)^a^	P-value^b^	RR (95% CI)	aRR^c^ (95% CI)
	n	n (%)			
**Sex**					
Male	37	13 (35.1)	0.37	REF	REF
Female	108	47 (43.5)		1.24 (0.76-2.02)	1.40 (0.87-2.27)
**Age group (in years)**					
18–49	74	25 (33.8)	0.16	REF	REF
50–64	50	25 (50.0)		1.48 (0.97-2.26)	1.35 (0.88-2.08)
≥65	21	10 (47.6)		1.41 (0.81-2.45)	1.32 (0.80-2.18)
**Enrollment site (service unit)**					
Chinle	81	33 (40.7)	0.68	REF	Not included
Tuba City	41	16 (39.0)		0.96 (0.60-1.53)	
Whiteriver	22	11 (50.0)		1.23 (0.75-2.01)	
**Education level** ^d^					
Some high school or less	10	6 (60.0)	0.33	REF	Not included
High school diploma or GED	51	18 (35.3)		0.59 (0.31-1.10)	
Some college or AA	63	24 (38.1)		0.63 (0.35-1.15)	
Completed degree, including graduate	17	9 (52.9)		0.88 (0.45-1.74)	
**Wood used to heat home**					
No	53	20 (37.7)	0.50	REF	Not included
Yes	92	40 (43.5)		1.15 (0.76-1.75)	
**Running water in home** ^e^					
No	23	8 (34.8)	0.47	0.81 (0.45-1.48)	Not included
Yes	119	51 (42.9)		REF	
**Presence of underlying medical condition**					
None	42	12 (28.6)	**0.05** ^k^	REF	REF
≥1^f^	103	48 (46.6)		1.63 (0.97-2.75)	1.53 (0.91-2.58)
**Medical presentation at time of acute illness** ^g^					
Outpatient	130	50 (38.5)	**0.04**	REF	REF
Inpatient	15	10 (66.7)		**1.73 (1.14-2.64)**	1.46 (0.96-2.20)
**Number of symptoms during acute illness**					
5 or less^h^	63	23 (36.5)	0.30	REF	Not included
6 or more	82	37 (45.1)		1.24 (0.82-1.85)	
**COVID-19 vaccination status at time of acute illness**					
Unvaccinated	14	9 (64.3)	0.07	REF	REF
Completed primary series^i^	131	51 (38.9)		**0.61 (0.39-0.95)**	**0.56 (0.34-0.90)**
**Self-reported and/or serologic evidence of prior SARS-CoV-2 infection** ^j^					
No	42	15 (35.7)	0.29	REF	Not included
Yes	63	29 (46.0)		1.29 (0.79-2.10)	

AA, Associate of Arts degree; CI, confidence interval; GED, General Educational Development; IQR, interquartile range; PCC, Post-COVID Conditions; RR, risk ratio; aRR, adjusted risk ratio; REF, reference category.

Note: PCC was defined as the presence of any self-reported symptom (new or persistent) thought to be related to the acute illness, any signs and symptoms of COVID-19 recorded in the EHR at the 3-month visit, or any new diagnoses recorded in the EHR at the 3-month visit. This analysis was restricted to adult participants who were enrolled during Omicron variant predominance and completed a 3-month interview. Omicron variant predominance was defined as the period during which Omicron was detected in >50% of sequenced cases using national trends [25], and corroborated by viral genomes sequenced as part of the current study, and occurred from December 25, 2021 onwards. Children were excluded from the risk factor analysis because of small sample size (n=11 with PCC). **Boldface** indicates statistical significance (defined as p-value < 0.05 or 95% CI that did not include 1.00).

^a^Row percentage indicating proportion that developed PCC among each covariate level.

^b^Differences in proportions for categorical variables estimated using Pearson *Χ*^2^ test or Fischer’s exact test when appropriate.

^c^Final model adjusted for age group, sex, presence of underlying medical condition, medical presentation at time of acute illness, and vaccination status at time of acute illness. Covariates included in final model were selected based on statistical significance in bivariable analyses and those established as risk factors in previous studies (i.e., regardless of statistical significance in bivariable analyses); model parsimony was also considered in selecting final covariates.

^d^Four (2.8%) missing education level.

^e^Three (2.1%) missing running water in home.

^f^Indicates participant has at least one of the following listed in their medical record: anxiety, asthma, chronic lung disease, depression, diabetes type 1 or 2, heart conditions (excluding hypertension only, included [but not limited to] atherosclerotic cardiovascular disease, cardiomyopathy, congestive heart failure, and/or coronary artery disease), hypertension, immunosuppression (due to treatment/therapy, bone marrow or solid organ transplant recipient), and/or obesity.

^g^Outpatient = participants enrolled at outpatient clinics, Emergency Departments, or SARS-CoV-2 testing clinics.

^h^4 adults had an asymptomatic acute illness; no adults with an asymptomatic acute illness had PCC.

^i^Completed primary series = Received two doses of an approved mRNA COVID-19 vaccine primary series or one dose of an approved non-mRNA vaccine ≥14 days prior to illness onset. May or may not have received ≥1 booster dose.

^j^Serologic evidence of prior infection determined by blood specimen positive for nucleocapsid IgG antibody; only blood specimens collected within 1 week of illness onset included (total n with serologic evidence within 1 week=125). Forty (27.8%) missing self-reported prior infection and/or serologic evidence.

^k^Rounded value. P-value=0.046.

Among adults whose acute illness occurred during Omicron predominance (n=145), a higher proportion of those who were older (50-64 and ≥65 years), had certain underlying medical conditions (S9 Table), were admitted to the hospital for their acute illness, and were unvaccinated, had PCC at three months (Table 4). In the final multivariable risk factor analysis, COVID-19 vaccination was significantly associated with a decreased risk of PCC (aRR: 0.56 [0.34–0.90]). Female sex (aRR: 1.40 [95% CI: 0.87–2.27]), having certain underlying medical conditions (aRR: 1.53 [0.91–2.58]), older age (50–64 years aRR: 1.35 [0.88–2.08]; ≥65 years aRR: 1.32 [0.80–2.18]), and hospital admission (aRR: 1.46 [0.96–2.20]) appeared to be associated with an increased risk of PCC, but the results were not statistically significant (Table 4). Results were similar when the analysis was repeated with the definition of PCC restricted to those with self-reported symptoms only (S10 Table).

Sixty-nine children had complete information from their 3-month visit and were assessed for PCC. Overall, 15.9% (11/69) of children had symptoms consistent with PCC: 14.8% (9/61) of children who presented as outpatients compared to 25.0% (2/8) of children who presented as inpatients (p-value=0.46), and 14.3% (1/7) of children initially asymptomatic during acute illness compared to 16.1% (10/62) of symptomatic children (p-value=1.00). The most common manifestations of PCC among children were cough (72.7%, 8/11) and runny nose (36.4%, 4/11); 27.3% (3/11) of children with PCC experienced at least three symptoms at 3 months (Fig 1). Symptoms via self-report, signs and symptoms from the EHR, and new conditions among children are reported in S5-S7 Tables, respectively. Children had few signs or symptoms by self-report or EHR, and very few new conditions. Because of the small number enrolled, risk factors for PCC were not assessed among children.

## Discussion

Among a cohort of individuals with predominantly mild COVID-19 from three rural Indigenous communities, 39.8% (86/216) of adults and 15.9% (11/69) of children exhibited signs and/or symptoms of PCC three months post-acute illness. Fatigue and/or tiredness, headache, and cough were the most common PCC symptoms among adults; among children, cough and runny nose were the most common.

The proportion of adults with symptoms of PCC three months after acute illness in this Indigenous cohort was consistent with reports from other studies conducted in the general population around the world with similar follow-up periods. In a recent multi-center study in the US with a similar study population (inpatients, 14.1%), 40.8% of participants reported symptoms after 3 months [28]. Although sample size was limited in this study, the proportion of inpatient adults with symptoms of PCC was 63.6%, consistent with results ranging from 48.5-66.7% with PCC in other studies that predominantly or exclusively included hospitalized participants (79–100%) [10,29]. Furthermore, the proportion of adults with symptoms of PCC among outpatients (37.1%) was similar to a recent systematic review of PCC among adults with mild acute illness (i.e., among those not hospitalized) in Europe and the US, that reported a prevalence of PCC of 10–35% [30]. Among children in the current study, 15.9% had symptoms consistent with PCC. Acute COVID-19 in children is generally mild and children are less likely to be hospitalized for COVID-19 compared to adults. PCC is less consistently documented among children compared to adults, with estimates in the literature ranging from <2–61% [31–35].

The most common manifestations of PCC among adults in the current study were fatigue/tiredness, headache, cough, shortness of breath, and myalgia, while cough and runny nose were commonest among children. This is consistent with previous studies in the general population — including a recent globally-focused systematic review of studies conducted three months post-acute illness —that have shown that fatigue with or without bodily pain or mood swings, “brain fog” (or, cognitive impairment), and respiratory symptoms are most often reported among patients with PCC [8–10]. Consistent with other studies, anxiety and depression and/or suicidal thoughts were observed among both adults and children, although the number was smaller than previously reported [36–38]. Despite the small proportion of study participants reporting these symptoms, these results may be useful in identifying needs and informing the continuum of COVID-19 care in study communities.

Among factors evaluated, only COVID-19 vaccination was significantly associated with a reduced risk of PCC. The effectiveness of COVID-19 vaccines in reducing the risk of severe disease is well established [19,39]. Similarly, reduced risk of PCC has been reported among persons vaccinated with a COVID-19 primary series prior to their acute illness as compared to those who were unvaccinated [40–43]. Although PCC can occur in persons with mild acute illness, the risk of PCC has been shown to increase with acute illness severity [9,44,45]. Therefore, it is reasonable to hypothesize that in reducing the risk of outcomes such as hospitalization and ICU admission, vaccination reduces the risk of downstream effects (i.e., PCC) as well. In addition, one recent meta-analysis found that at least two doses of COVID-19 vaccine were protective against PCC, even among persons who received the complete series after their acute illness [43]. The current study did not consider vaccinations received after the acute phase. However, consistent with previous findings, vaccination with at least a completed primary series prior to the acute illness was strongly protective against PCC among adults. This underscores the importance of COVID-19 vaccination in reducing the risk of morbidity and mortality, especially among disproportionately impacted populations such as Indigenous communities.

While female sex, older age, presence of underlying conditions and severity of the acute SARS-CoV-2 infection among this Indigenous cohort were not significantly associated with PCC, the trends for these variables were consistent with other studies from the general population [8,9,11,44–49]. In the current study, female sex was marginally associated with increased risk of PCC. Differences in immune system function between males and females are well documented [50,51], which may influence sex differences in PCC. However, behavioral drivers should also be considered. It has been shown consistently that males are less likely to seek healthcare compared to females [52,53], potentially leading to under ascertainment of PCC among males. Increased risk of PCC was indicated among adults with asthma, hypertension, and immunosuppression in this analysis. Understanding associations between underlying medical conditions and PCC will be critical for chronic disease management, particularly in populations with high burden of chronic disease (e.g., hypertension), such as Indigenous persons, as they are potentially vulnerable to synergistic consequences of PCC.

The definition of PCC differs between organizations (e.g., WHO versus CDC; see S1 Table), and even terminology to describe the condition is inconsistent across studies (e.g., PCC, PASC, Long COVID). The lack of a standardized definition or validated clinical guidelines for its diagnosis contributes to challenges in characterizing prevalence, symptoms, and associated risk factors. This also makes comparing our results to previous studies challenging. For example, although one previous study evaluated hospitalized patients using the same 3-month follow-up period as the current study [29], the study used the WHO definition of PCC, which requires a negative impact on function and for the symptoms to be present for at least two months [7]. The definition used for the current study only required symptoms to be reported by the participant or documented in the medical record, which may lead to higher estimates of PCC. Other studies did not include self-reported symptoms and instead relied on ICD codes [44,54], which requires the person to have sought care, or evaluated symptoms over a longer follow-up period (e.g., six or 12 months) [55,56], both of which may lead to lower estimates of PCC. Furthermore, the lack of previous studies conducted among underserved populations, especially Indigenous communities, further complicates our ability to contextualize these results. To our knowledge, only one other study has reported results from an Indigenous community in Wisconsin. In that study, which was conducted predominantly among adults (70%) and individuals with mild illness (96% symptomatic and 6% hospitalized), 22% met the definition of an “ever long hauler” [16]. Challenges accessing healthcare services in rural settings may bar many Indigenous persons with PCC from seeking appropriate care, or from being diagnosed with PCC at all. Given the disproportionate burden of COVID-19 experienced by Indigenous communities and increased prevalence of risk factors for both severe illness and PCC, additional representation of Indigenous populations in COVID-19 research is needed.

This analysis is subject to several limitations. Study recruitment was biased towards symptomatic individuals – less than 5% of all participants were asymptomatic at the time of their acute illness – which limited our ability to assess the full spectrum of SARS-CoV-2 infection. The description of symptoms and outcomes during the acute illness should be interpreted within the context of symptomatic persons presenting for medical care or testing and should not be interpreted as a clinical description of SARS-CoV-2 infection in this population or Indigenous persons more broadly. The small sample size, particularly of hospitalized patients and children, limited our ability to detect associations between patient characteristics and occurrence of PCC. Because of challenges accessing patient rooms and safety considerations during the pandemic, collection of research specimens was often delayed relative to participants’ presentation for care, which limited our ability to document serologic evidence of prior infection; prior infection status by self-report or serology was missing for a quarter of adults and half of children participating in the study. Prior infection has been shown to attenuate the effectiveness of COVID-19 booster doses compared to those without prior infection [57,58]. Thus, our ability to assess independent and/or synergistic effects of prior infection and vaccination status on risk of progression to PCC was limited. Furthermore, previous studies have demonstrated that nucleocapsid seroconversion may occur earlier than seven days, so evidence of prior infection may have been overestimated among those whose prior infection status was determined by serology, as presence of anti-nucleocapsid antibodies for some participants may have been the result of their acute illness [59]. We were not able to assess duration or severity of PCC symptoms. However, our characterization of PCC is robust because of the use of self-reported and clinically observed symptoms, as well as new documented medical conditions. These signs, symptoms, and conditions included in our definition are comprehensive and comparable to those used to classify PCC by CDC, WHO, and other COVID-19 research initiatives (e.g., ISARIC). An additional limitation is the lack of a comparison group, which limits our ability to assess the degree to which signs and symptoms were attributable to PCC. Previous studies that have included non-SARS-CoV-2 control groups have shown significantly higher rates of symptoms post-acute illness among SARS-CoV-2 cases compared to controls [55,60]; although other studies have demonstrated that ongoing symptoms and new diagnoses also follow other viral infections [61]. As many of the signs and symptoms are non-specific, there is also the possibility they were caused by new acute infections during follow-up. The challenges of selecting an appropriate control group in future studies should be carefully considered.

## Conclusions

Indigenous populations have experienced a disproportionate COVID-19 burden but have been underrepresented in studies evaluating its long-term effects. Results from these analyses fill a critical data gap and show a significant proportion of Indigenous persons in the Southwest US have symptoms consistent with PCC three months following acute illness, including among individuals with mild acute illness. These results highlight the importance of allocating resources to support referral services in rural and other underserved communities. The inclusion of screening for PCC and referrals for chronic disease management in clinical care pathways, delivered in culturally competent formats, are urgently needed to support wellness and inform the continuum of COVID-19 care.

## Supporting information

S1 Checklist Inclusivity in global research. (DOCX)

S1 Table Post-COVID-19 Conditions definitions: current study, CDC, WHO, and select studies. (DOCX)

S2 Table Signs and symptoms experienced by adults during acute illness, by symptom ascertainment and medical presentation. (DOCX)

S3 Table Signs and symptoms experienced by children during acute illness, by symptom ascertainment and medical presentation. (DOCX)

S4 Table Symptoms self-reported and/or documented in the electronic health record during the acute COVID-19 illness, by SARS-CoV-2 variant predominance and age group, among outpatient participants. (DOCX)

S5 Table Characteristics of adult participants with 3-month follow up data, overall and by variant period. (DOCX)

S6 Table Self-reported symptoms three months post-acute illness, by age and medical presentation. (DOCX)

S7 Table Signs and symptoms recorded in EHR three months post-acute illness, by age and medical presentation. (DOCX)

S8 Table New conditions recorded in EHR three months post-acute illness, by age and medical presentation. (DOCX)

S9 Table Selected underlying medical conditions associated with PCC at three months post-acute illness among adults. (DOCX)

S10 Table Sociodemographic and clinical characteristics associated with PCC at three months post-acute illness among adults enrolled during Omicron predominance– definition of PCC restricted to self-reported symptoms. (DOCX)

S1 Fig Flow diagram of participant enrollment and completed study activities. (DOCX)

S2 Fig Antibody positivity among participants who had a blood specimen collected <1 week after onset of acute illness, by age group, COVID-19 vaccination status, and target protein. (DOCX)

S3 Fig SARS-CoV-2 sequencing results for specimens collected during acute illness, by month of swab collection. (DOCX)
