# Supplementary material for: Clinical characterization of acute COVID-19 and Post-COVID-19 Conditions 3 months following infection: A cohort study among Indigenous adults and children in the Southwestern United States
Source: PLOS Glob Public Health. 2025 Mar 18;5(3):e0004204. doi: 10.1371/journal.pgph.0004204 (PMC11918431; doi:10.1371/journal.pgph.0004204)
Supplement: S1 Table — (DOCX) [file pgph.0004204.s002.docx]

| **S1 Table. Post-COVID-19 Conditions definitions: current study, CDC, WHO, and select studies** | | | | | |
| --- | --- | --- | --- | --- | --- |
| **Current analysis** | **Clinical case definitions** | | **Research case definitions** | | |
|  | **CDC^a^** | **WHO^b^** | **ISARIC^c^** | **RECOVER^d^** | **INSPIRE^e^** |
| Presence of any self-reported symptom (new or persistent) that the participant thought to be related to their acute COVID-19 illness at the 3-month visit, any signs and symptoms of COVID-19 recorded in the EHR after the acute illness and up to the 3-month visit, and/or new report of one of the following medical conditions in the EHR after the acute illness and up to the 3-month visit. | Signs, symptoms, and conditions that continue or develop after initial SARS-CoV-2 infection. The signs, symptoms, and conditions are present four weeks or more after the initial phase of infection. | Post COVID-19 condition occurs in individuals with a history of probable or confirmed SARS-CoV-2 infection, usually 3 months from onset of COVID-19 with symptoms that last for ≥2 months and can’t be explained by an alternative diagnosis. Symptoms generally have an impact on everyday functioning. | Physical and psychosocial consequences in patients post-acute COVID- 19, at least one month after hospital discharge. | Ongoing, relapsing, or new symptoms, or other health effects occurring after the acute phase of SARS-CoV-2 infection (i.e., present four or more weeks after the acute infection). | Medium- and long-term sequelae of symptomatic SARS-CoV-2 infection. Analyses of persistent or emergent symptoms 3 and 6 months after acute illness have been performed. |
| ^a^Reference: Centers for Disease Control and Prevention. Long COVID or Post-COVID Conditions 2023 [Available from: https://www.cdc.gov/coronavirus/2019-ncov/long-term-effects/index.html] | | | | | |
| ^b^Reference: World Health Organization. A clinical case definition of post COVID-19 condition by a Delphi consensus, 6 October 2021 2021 Oct 6 [Available from: <https://www.who.int/publications/i/item/WHO-2019-nCoV-Post_COVID-19_condition-Clinical_case_definition-2021.1>.] | | | | | |
| ^c^Reference: International Severe Acute Respiratory and emerging Infection Consortium. COVID-19 Health and Wellbeing Follow Up Survey 2021 [Available from: <https://isaric.org/wp-content/uploads/2021/03/Initial-Freestanding-survey.pdf>.] | | | | | |
| ^d^Reference: Reese JT, Blau H, Casiraghi E, et al.; N3C Consortium; RECOVER Consortium. Generalisable long COVID subtypes: findings from the NIH N3C and RECOVER programmes. EBioMedicine. 2023 Jan;87:104413. doi: 10.1016/j.ebiom.2022.104413. Information about the RECOVER Initiative available at: <https://recovercovid.org/>. | | | | | |
| ^e^References: 1) O'Laughlin KN, Thompson M, Hota B, et al; INSPIRE Investigators. Study protocol for the Innovative Support for Patients with SARS-COV-2 Infections Registry (INSPIRE): A longitudinal study of the medium and long-term sequelae of SARS-CoV-2 infection. PLoS One. 2022 Mar 3;17(3):e0264260. doi: 10.1371/journal.pone.0264260.  2) Spatz ES, Gottlieb M, Wisk LE, et al. Three-Month Symptom Profiles Among Symptomatic Adults With Positive and Negative Severe Acute Respiratory Syndrome Coronavirus 2 Tests: A Prospective Cohort Study From the INSPIRE Group. Clin Infect Dis. 2023 May 3;76(9):1559-1566. doi: 10.1093/cid/ciac966.  3) Gottlieb M, Spatz ES, Yu H, et al; INSPIRE Group. Long COVID Clinical Phenotypes up to 6 Months After Infection Identified by Latent Class Analysis of Self-Reported Symptoms. Open Forum Infect Dis. 2023 May 31;10(7):ofad277. doi: 10.1093/ofid/ofad277. | | | | | |
