# Supplementary material for: Clinical characterization of acute COVID-19 and Post-COVID-19 Conditions 3 months following infection: A cohort study among Indigenous adults and children in the Southwestern United States
Source: PLOS Glob Public Health. 2025 Mar 18;5(3):e0004204. doi: 10.1371/journal.pgph.0004204 (PMC11918431; doi:10.1371/journal.pgph.0004204)
Supplement: S2 Table — (DOCX) [file pgph.0004204.s003.docx]

| **S2 Table. Signs and symptoms experienced by adults during acute illness, by symptom ascertainment and medical presentation** | | | | | | |
| --- | --- | --- | --- | --- | --- | --- |
|  | **Self-reported** | | | **Documented in EHR** | | |
|  | **Total^a^ (N=251)** | **Inpatient (n=35)** | **Outpatient (n=216)** | **Total^a^ (N=254)** | **Inpatient (n=38)** | **Outpatient (n=216)** |
|  | **n (%)** | **n (%)** | **n (%)** | **n (%)** | **n (%)** | **n (%)** |
| **Systemic** | 161 (64.1) | 24 (68.6) | 137 (63.4) | 129 (50.8) | 30 (78.9) | 99 (45.8) |
| Chills/rigors | 96 (38.2) | 18 (51.4) | 78 (36.1) | 35 (13.8) | 8 (21.1) | 27 (12.5) |
| Difficulty sleeping | NA | NA | NA | 1 (0.4) | 0 (0.0) | 1 (0.5) |
| Fatigue/tiredness^b^ | 98 (39.0) | 18 (51.4) | 80 (37.0) | 12 (4.7) | 3 (7.9) | 9 (4.2) |
| Fever | 88 (35.1) | 16 (45.7) | 72 (33.3) | 51 (20.1) | 19 (50.0) | 32 (14.8) |
| Malaise | NA | NA | NA | 4 (1.6) | 0 (0.0) | 4 (1.9) |
| Sepsis or shock | NA | NA | NA | 0 (0.0) | 0 (0.0) | 0 (0.0) |
| Weak/dizzy | 75 (29.9) | 13 (37.1) | 62 (28.7) | 14 (5.5) | 6 (15.8) | 8 (3.7) |
|  |  |  |  |  |  |  |
| **Respiratory** | 190 (75.7) | 28 (80.0) | 162 (75.0) | 190 (74.8) | 28 (73.7) | 162 (75.0) |
| Acute respiratory distress | NA | NA | NA | 0 (0.0) | 0 (0.0) | 0 (0.0) |
| Apnea | NA | NA | NA | 0 (0.0) | 0 (0.0) | 0 (0.0) |
| Chest pain/tightness | 41 (16.3) | 10 (28.6) | 31 (14.4) | 14 (5.5) | 6 (15.8) | 8 (3.7) |
| Cough | 172 (68.5) | 24 (68.6) | 148 (68.5) | 170 (66.9) | 34 (89.5) | 136 (63.0) |
| Decreased breathing sounds | NA | NA | NA | 6 (2.4) | 5 (13.2) | 1 (0.5) |
| Pain when coughing | NA | NA | NA | 1 (0.4) | 0 (0.0) | 1 (0.5) |
| Pneumonia | NA | NA | NA | 8 (3.1) | 6 (15.8) | 2 (0.9) |
| Rales | NA | NA | NA | 9 (3.5) | 9 (23.7) | 0 (0.0) |
| Respiratory distress | NA | NA | NA | 2 (0.8) | 2 (5.3) | 0 (0.0) |
| Retractions | NA | NA | NA | 0 (0.0) | 0 (0.0) | 0 (0.0) |
| Shortness of breath | 70 (27.9) | 18 (51.4) | 52 (23.6) | 45 (17.7) | 25 (65.8) | 20 (9.3) |
| Sputum production | 69 (27.5) | 14 (40.0) | 55 (25.5) | 13 (2.0) | 8 (7.9) | 5 (2.3) |
| Stridor | NA | NA | NA | 1 (0.4) | 1 (2.6) | 0 (0.0) |
| Tachypnea | NA | NA | NA | 4 (1.6) | 4 (10.5) | 0 (0.0) |
| Wheeze | 54 (21.5) | 16 (45.7) | 38 (17.6) | 5 (2.0) | 3 (7.9) | 2 (0.9) |
|  |  |  |  |  |  |  |
| **Head, ear, nose, throat** | 222 (88.4) | 26 (74.3) | 196 (90.7) | 222 (87.4) | 26 (68.4) | 196 (90.7) |
| Congestion | 102 (40.6) | 10 (28.6) | 92 (42.6) | 14 (5.5) | 1 (2.6) | 13 (6.0) |
| Conjunctivitis | 25 (10.0) | 6 (17.1) | 19 (8.8) | 1 (0.4) | 0 (0.0) | 1 (0.5) |
| Ear pain | NA | NA | NA | 10 (3.9) | 0 (0.0) | 10 (4.6) |
| Headache | 156 (62.2) | 21 (60.0) | 135 (62.5) | 102 (40.2) | 12 (31.6) | 90 (41.7) |
| Runny nose | 146 (58.2) | 17 (48.6) | 129 (59.7) | 146 (57.5) | 17 (44.7) | 129 (59.7) |
| Sinus pain | NA | NA | NA | 4 (1.6) | 0 (0.0) | 4 (1.9) |
| Sneezing | NA | NA | NA | 1 (0.4) | 0 (0.0) | 1 (0.5) |
| Sore/itchy throat | 123 (49.0) | 12 (34.3) | 111 (51.4) | 124 (48.8) | 12 (31.6) | 112 (51.9) |
|  |  |  |  |  |  |  |
| **Neurologic** | 66 (26.3) | 9 (25.7) | 57 (26.4) | 25 (9.8) | 5 (13.2) | 20 (9.3) |
| Loss of taste or smell | 66 (26.3) | 9 (25.7) | 57 (26.4) | 24 (9.4) | 4 (10.5) | 20 (9.3) |
| Seizure | NA | NA | NA | 1 (0.4) | 1 (2.6) | 0 (0.0) |
|  |  |  |  |  |  |  |
| **Mental acuity** |  |  |  |  |  |  |
| Confusion | 26 (10.4) | 8 (22.9) | 18 (8.3) | 26 (10.1) | 8 (21.1) | 18 (8.3) |
|  |  |  |  |  |  |  |
| **Circulatory** | NA | NA | NA | 9 (3.5) | 8 (21.1) | 1 (0.5) |
| Cyanosis | NA | NA | NA | 0 (0.0) | 0 (0.0) | 0 (0.0) |
| Hypoxemia | NA | NA | NA | 9 (3.5) | 8 (21.1) | 1 (0.5) |
|  |  |  |  |  |  |  |
| **Cardiac** |  |  |  |  |  |  |
| Tachycardic | NA | NA | NA | 5 (1.9) | 0 (0.0) | 5 (2.3) |
|  |  |  |  |  |  |  |
| **Gastrointestinal** | 100 (39.8) | 18 (51.4) | 82 (38.0) | 100 (38.8) | 18 (47.4) | 82 (38.0) |
| Abdominal pain | 18 (7.2) | 6 (17.1) | 12 (5.6) | 1 (0.4) | 0 (0.0) | 1 (0.5) |
| Diarrhea | 44 (17.5) | 13 (37.1) | 31 (14.4) | 8 (3.1) | 5 (13.2) | 3 (1.4) |
| Loss of appetite | 58 (23.1) | 12 (34.3) | 46 (21.3) | 58 (22.5) | 12 (31.6) | 46 (21.3) |
| Nausea | 52 (20.7) | 11 (31.4) | 41 (19.0) | 14 (5.4) | 6 (15.8) | 8 (3.7) |
| Transaminitis | NA | NA | NA | 1 (0.4) | 0 (0.0) | 1 (0.5) |
| Vomiting | 25 (10.0) | 10 (28.6) | 15 (6.9) | 25 (9.7) | 10 (26.3) | 15 (6.9) |
|  |  |  |  |  |  |  |
| **Musculoskeletal** | 125 (49.8) | 20 (57.1) | 105 (48.6) | 125 (48.4) | 20 (52.6) | 105 (48.6) |
| Muscle aches/myalgia | 125 (49.8) | 20 (57.1) | 105 (48.6) | 125 (48.4) | 20 (52.6) | 105 (48.6) |
| Red or bruised toes | 4 (1.6) | 2 (5.7) | 2 (0.9) | NA | NA | NA |
| Rash | NA | NA | NA | 1 (0.4) | 0 (0.0) | 1 (0.5) |
|  |  |  |  |  |  |  |
| **Other^c,d^** | 4 (1.6) | 1 (2.6) | 3 (1.4) | 14 (5.5) | 6 (15.8) | 8 (3.7) |
| EHR, Electronic health record; NA, not applicable (signs and symptoms listed as options in the EHR may differ from those asked about during participant interviews). | | | | | | |
| ^a^3 adults did not complete a baseline questionnaire and do not have self-reported symptoms. Four asymptomatic adults not included in counts. | | | | | | |
| ^b^Tiredness includes lethargy and abnormally sleepy. | | | | | | |
| ^c^Other self-reported included the following: watery eyes; intermittent burning sensation in hands and feet; thirsty (n=2) | | | | | | |
| ^d^Other documented in the EHR included the following: one episode of vomiting blood; leg swelling; red bump on left cheek; sweats; myalgia, possibly due to arthritis and not COVID-19; feels dehydrated; coughing up blood, bloody nose, muscle cramps; mild metabolic acidosis; anxiety attack; shoulder pain; tooth pain; “barely audible” expiration diffusion; allergies; light sensitivity, constipation. | | | | | | |
