# Supplementary material for: Clinical characterization of acute COVID-19 and Post-COVID-19 Conditions 3 months following infection: A cohort study among Indigenous adults and children in the Southwestern United States
Source: PLOS Glob Public Health. 2025 Mar 18;5(3):e0004204. doi: 10.1371/journal.pgph.0004204 (PMC11918431; doi:10.1371/journal.pgph.0004204)
Supplement: S3 Table — (DOCX) [file pgph.0004204.s004.docx]

| **S3 Table. Signs and symptoms experienced by children during acute illness, by symptom ascertainment and medical presentation** | | | | |
| --- | --- | --- | --- | --- |
|  | **Self-reported** | | **Documented in EHR** | |
|  | **Total^a^ (N=76)** | **Outpatient (n=68)** | **Total^a^ (N=76)** | **Outpatient (n=68)** |
|  | **n (%)** | **n (%)** | **n (%)** | **n (%)** |
| **Systemic** | 40 (52.6) | 35 (51.5) | 33 (43.4) | 27 (39.7) |
| Chills/rigors | 14 (16.7) | 14 (20.6) | 4 (5.3) | 4 (5.9) |
| Difficulty sleeping | NA | NA | 0 (0.0) | 0 (0.0) |
| Fever | 30 (39.5) | 25 (36.8) | 15 (19.7) | 11 (16.2) |
| Fatigue/tiredness^b^ | 9 (11.8) | 8 (11.8) | 2 (2.6) | 0 (0.0) |
| Malaise | NA | NA | 1 (1.3) | 1 (1.3) |
| Sepsis or shock | NA | NA | 0 (0.0) | 0 (0.0) |
| Weak/dizzy | 13 (17.1) | 12 (17.6) | 2 (2.6) | 2 (2.9) |
|  |  |  |  |  |
| **Respiratory** | 58 (76.3) | 51 (75.0) | 58 (76.3) | 51 (75.0) |
| Acute respiratory distress | NA | NA | 0 (0.0) | 0 (0.0) |
| Apnea | NA | NA | 0 (0.0) | 0 (0.0) |
| Chest pain/tightness | 7 (9.2) | 6 (8.8) | 0 (0.0) | 0 (0.0) |
| Cough | 53 (63.1) | 47 (69.1) | 38 (50.0) | 32 (47.1) |
| Decreased breathing sounds | NA | NA | 0 (0.0) | 0 (0.0) |
| Pain when coughing | NA | NA | 0 (0.0) | 0 (0.0) |
| Pneumonia | NA | NA | 0 (0.0) | 0 (0.0) |
| Rales | NA | NA | 0 (0.0) | 0 (0.0) |
| Respiratory distress | NA | NA | 1 (1.3) | 0 (0.0) |
| Retractions | NA | NA | 4 (5.3) | 0 (0.0) |
| Shortness of breath | 9 (11.8) | 6 (8.8) | 5 (6.6) | 1 (1.5) |
| Sputum production | 13 (17.1) | 11 (16.2) | 1 (1.3) | 0 (0.0) |
| Stridor | NA | NA | 1 (1.3) | 0 (0.0) |
| Tachypnea | NA | NA | 2 (2.6) | 0 (0.0) |
| Wheeze | 6 (7.9) | 4 (5.9) | 4 (5.3) | 2 (2.9) |
|  |  |  |  |  |
| **Head, ear, nose, throat** | 63 (82.9) | 57 (83.8) | 63 (82.9) | 57 (83.8) |
| Congestion | 12 (15.8) | 10 (14.7) | 4 (5.3) | 4 (5.9) |
| Conjunctivitis | 7 (9.2) | 4 (5.9) | 0 (0.0) | 0 (0.0) |
| Ear pain | NA | NA | 0 (0.0) | 0 (0.0) |
| Headache | 27 (35.5) | 26 (38.2) | 13 (17.1) | 13 (19.1) |
| Runny nose | 44 (57.9) | 40 (58.8) | 27 (35.5) | 23 (33.8) |
| Sinus pain | NA | NA | 0 (0.0) | 0 (0.0) |
| Sneezing | NA | NA | 0 (0.0) | 0 (0.0) |
| Sore/itchy throat | 31 (40.8) | 30 (44.1) | 25 (32.9) | 25 (36.8) |
|  |  |  |  |  |
| **Neurologic** | 16 (21.1) | 15 (22.1) | 4 (5.3) | 4 (5.9) |
| Loss of taste or smell | 16 (21.1) | 15 (22.1) | 3 (3.9) | 3 (4.4) |
| Seizure | NA | NA | 1 (1.3) | 1 (1.5) |
|  |  |  |  |  |
| **Mental acuity** |  |  |  |  |
| Confusion | 2 (2.6) | 2 (2.9) | 0 (0.0) | 0 (0.0) |
|  |  |  |  |  |
| **Circulatory** | NA | NA | 0 (0.0) | 0 (0.0) |
| Cyanosis | NA | NA | 0 (0.0) | 0 (0.0) |
| Hypoxemia | NA | NA | 0 (0.0) | 0 (0.0) |
|  |  |  |  |  |
| **Cardiac** |  |  |  |  |
| Tachycardic | NA | NA | 1 (1.3) | 1 (1.5) |
|  |  |  |  |  |
| **Gastrointestinal** | 31 (40.8) | 26 (38.2) | 31 (40.8) | 26 (38.2) |
| Abdominal pain | 1 (1.3) | 0 (0.0) | 0 (0.0) | 0 (0.0) |
| Diarrhea | 10 (13.2) | 9 (13.2) | 5 (6.6) | 5 (7.4) |
| Loss of appetite | 11 (14.5) | 9 (13.2) | 1 (1.3) | 0 (0.0) |
| Nausea | 11 (14.5) | 10 (14.7) | 2 (2.6) | 2 (2.9) |
| Vomiting | 16 (21.1) | 11 (16.2) | 5 (6.6) | 3 (4.4) |
|  |  |  |  |  |
| **Musculoskeletal** | 27 (35.5) | 26 (38.2) | 16 (21.1) | 16 (21.1) |
| Muscle aches/myalgia | 28 (36.8) | 27 (39.7) | 16 (21.1) | 16 (21.1) |
| Red or bruised toes | 0 (0.0) | 0 (0.0) | NA | NA |
| Rash | NA | NA | 1 (1.3) | 1 (1.5) |
|  |  |  |  |  |
| **Other** | 0 (0.0) | 0 (0.0) | 0 (0.0) | 0 (0.0) |
| EHR, electronic health record; NA, not applicable (signs and symptoms listed as options in the EHR may differ from those asked about during participant interviews). | | | | |
| ^a^Eight asymptomatic children not included in counts. Data on inpatient children not presented because of sparse data (N<10). | | | | |
| ^b^Tiredness includes lethargy and abnormally sleepy | | | | |
