# Supplementary material for: Clinical characterization of acute COVID-19 and Post-COVID-19 Conditions 3 months following infection: A cohort study among Indigenous adults and children in the Southwestern United States
Source: PLOS Glob Public Health. 2025 Mar 18;5(3):e0004204. doi: 10.1371/journal.pgph.0004204 (PMC11918431; doi:10.1371/journal.pgph.0004204)
Supplement: S4 Table — (DOCX) [file pgph.0004204.s005.docx]

| **S4 Table. Symptoms self-reported and/or documented in the electronic health record during the acute COVID-19 illness, by SARS-CoV-2 variant predominance and age group, among outpatient participants** | | | | | | | | |
| --- | --- | --- | --- | --- | --- | --- | --- | --- |
|  | **Adults (≥18 years)** | | | | **Children (<18 years)** | | | |
|  | **Total (N=216)**^a^ | **Pre-Omicron (n=74)** | **Omicron (n=142)** | **p-value^b^** | **Total (N=68)**^a^ | **Pre-Omicron (n=23)** | **Omicron (n=45)** | **p-value^b^** |
|  | **n (%)** | **n (%)** | **n (%)** |  | **n (%)** | **n (%)** | **n (%)** |  |
| **Systemic** | 170 (78.7) | 57 (77.0) | 113 (79.6) | 0.66 | 46 (67.6) | 12 (52.2) | 34 (75.6) | 0.06 |
| Chills/rigors | 89 (41.2) | 31 (41.9) | 58 (40.8) | 0.88 | 17 (25.0) | 2 (8.7) | 15 (33.3) | **0.04** |
| Difficulty sleeping | 1 (0.5) | 1 (1.3) | 0 (0.0) | 0.35 | 0 (0.0) | 0 (0.0) | 0 (0.0) | NA |
| Fatigue and/or tiredness^c^ | 83 (38.4) | 28 (37.8) | 55 (38.7) | 0.90 | 8 (11.8) | 1 (4.3) | 7 (15.6) | 0.25 |
| Fever | 88 (40.7) | 31 (41.9) | 57 (40.1) | 0.80 | 30 (44.1) | 6 (26.1) | 24 (53.3) | **0.04** |
| Malaise | 4 (1.9) | 1 (1.3) | 3 (2.1) | 1.00 | 1 (1.5) | 1 (3.3) | 0 (0.0) | 0.34 |
| Sepsis or shock | 0 (0.0) | 0 (0.0) | 0 (0.0) | NA | 0 (0.0) | 0 (0.0) | 0 (0.0) | NA |
| Weak/dizzy | 67 (31.0) | 25 (33.8) | 42 (30.0) | 0.53 | 12 (17.6) | 1 (4.3) | 11 (24.4) | **0.04** |
|  |  |  |  |  |  |  |  |  |
| **Respiratory** | 162 (75.0) | 51 (68.9) | 111 (78.2) | 0.14 | 51 (75.0) | 12 (52.2) | 39 (86.7) | **<0.01** |
| Acute respiratory distress | 0 (0.0) | 0 (0.0) | 0 (0.0) | NA | 0 (0.0) | 0 (0.0) | 0 (0.0) | NA |
| Apnea | 0 (0.0) | 0 (0.0) | 0 (0.0) | NA | 0 (0.0) | 0 (0.0) | 0 (0.0) | NA |
| Chest pain and/or tightness | 32 (14.8) | 12 (16.2) | 20 (14.1) | 0.68 | 6 (8.8) | 0 (0.0) | 6 (13.3) | 0.09 |
| Cough | 180 (83.3) | 59 (79.7) | 121 (85.2) | 0.31 | 51 (75.0) | 13 (56.5) | 38 (84.4) | **0.02** |
| Decreased breathing sounds | 1 (0.5) | 1 (1.3) | 0 (0.0) | 0.35 | 0 (0.0) | 0 (0.0) | 0 (0.0) | NA |
| Pain when coughing | 1 (0.5) | 1 (1.3) | 0 (0.0) | 0.35 | 0 (0.0) | 0 (0.0) | 0 (0.0) | NA |
| Pneumonia | 2 (0.9) | 2 (2.7) | 0 (0.0) | **0.05^e^** | 0 (0.0) | 0 (0.0) | 0 (0.0) | NA |
| Rales | 0 (0.0) | 0 (0.0) | 0 (0.0) | NA | 0 (0.0) | 0 (0.0) | 0 (0.0) | NA |
| Respiratory distress | 0 (0.0) | 0 (0.0) | 0 (0.0) | NA | 0 (0.0) | 0 (0.0) | 0 (0.0) | NA |
| Retractions | 0 (0.0) | 0 (0.0) | 0 (0.0) | NA | 0 (0.0) | 0 (0.0) | 0 (0.0) | NA |
| Shortness of breath | 63 (29.2) | 25 (33.8) | 38 (26.8) | 0.28 | 7 (10.3) | 3 (13.0) | 4 (8.9) | 0.68 |
| Sputum production | 57 (26.4) | 17 (23.0) | 40 (28.2) | 0.41 | 11 (16.2) | 0 (0.0) | 11 (24.4) | **0.01** |
| Stridor | 0 (0.0) | 0 (0.0) | 0 (0.0) | NA | 0 (0.0) | 0 (0.0) | 0 (0.0) | NA |
| Tachypnea | 0 (0.0) | 0 (0.0) | 0 (0.0) | NA | 0 (0.0) | 0 (0.0) | 0 (0.0) | NA |
| Wheeze | 39 (18.1) | 10 (13.5) | 29 (20.4) | 0.21 | 6 (8.8) | 0 (0.0) | 6 (13.3) | 0.09 |
|  |  |  |  |  |  |  |  |  |
| **Head, ear, nose, throat** | 196 (90.7) | 69 (93.2) | 127 (89.4) | 0.36 | 57 (83.8) | 17 (73.9) | 40 (88.9) | 0.11 |
| Congestion | 95 (44.0) | 28 (37.8) | 67 (47.2) | 0.19 | 13 (25.0) | 2 (8.7) | 11 (24.4) | 0.19 |
| Conjunctivitis | 19 (8.8) | 6 (8.1) | 13 (9.2) | 0.78 | 4 (5.9) | 1 (4.3) | 3 (6.7) | 1.00 |
| Ear pain | 10 (4.6) | 1 (1.3) | 9 (6.2) | 0.17 | 0 (0.0) | 10 (43.5) | 21 (46.7) | NA |
| Headache | 155 (71.8) | 53 (71.6) | 102 (71.8) | 0.97 | 31 (45.6) | 10 (43.5) | 21 (46.7) | 0.80 |
| Sinus pain | 4 (1.9) | 1 (1.3) | 3 (2.1) | 1.00 | 0 (0.0) | 0 (0.0) | 0 (0.0) | NA |
| Runny nose | 129 (59.7) | 43 (58.1) | 86 (60.6) | 0.73 | 0 (0.0) | 14 (60.9) | 33 (73.3) | 0.29 |
| Sneezing | 1 (0.5) | 1 (1.3) | 0 (0.0) | 0.35 | 0 (0.0) | 0 (0.0) | 0 (0.0) | NA |
| Sore/itchy throat | 112 (51.9) | 24 (32.4) | 88 (62.0) | **<0.01** | 38 (55.9) | 9 (39.1) | 29 (64.4) | **0.05^f^** |
|  |  |  |  |  |  |  |  |  |
| **Cognitive** |  |  |  |  |  |  |  |  |
| Confusion | 18 (8.3) | 6 (8.1) | 12 (8.5) | 0.93 | 2 (2.9) | 0 (0.0) | 2 (4.4) | 0.55 |
|  |  |  |  |  |  |  |  |  |
| **Neurologic** | 63 (29.2) | 31 (41.9) | 32 (22.5) | **<0.01** | 18 (265) | 6 (26.1) | 12 (26.7) | 0.96 |
| Loss of taste or smell | 63 (29.2) | 31 (41.9) | 32 (22.5) | **<0.01** | 17 (25.0) | 6 (26.1) | 11 (24.4) | 0.88 |
| Seizure | 0 (0.0) | 0 (0.0) | 0 (0.0) | NA | 1 (1.5) | 0 (0.0) | 1 (2.2) | 1.00 |
|  |  |  |  |  |  |  |  |  |
| **Circulatory** | 1 (0.5) | 1 (1.3) | 0 (0.0) | 0.35 | 0 (0.0) | 0 (0.0) | 0 (0.0) | NA |
| Cyanosis | 0 (0.0) | 0 (0.0) | 0 (0.0) | NA | 0 (0.0) | 0 (0.0) | 0 (0.0) | NA |
| Hypoxemia | 1 (0.5) | 1 (1.3) | 0 (0.0) | 0.35 | 0 (0.0) | 0 (0.0) | 0 (0.0) | NA |
|  |  |  |  |  |  |  |  |  |
| **Cardiac** |  |  |  |  |  |  |  |  |
| Tachycardia | 5 (2.3) | 4 (5.3) | 1 (0.7) | **0.05^g^** | 1 (1.5) | 0 (0.0) | 1 (2.2) | 1.00 |
|  |  |  |  |  |  |  |  |  |
| **Gastrointestinal** | 82 (38.0) | 28 (37.8) | 54 (38.0) | 0.98 | 26 (38.2) | 6 (26.1) | 20 (44.4) | 0.14 |
| Abdominal/stomach pain | 13 (6.0) | 3 (4.1) | 10 (7.0) | 0.55 | 0 (0.0) | 0 (0.0) | 0 (0.0) | NA |
| Diarrhea | 33 (15.3) | 11 (14.9) | 22 (15.5) | 0.90 | 11 (16.2) | 2 (8.7) | 9 (20.0) | 0.31 |
| Loss of appetite | 46 (21.3) | 20 (27.0) | 26 (18.3) | 0.14 | 9 (13.2) | 3 (13.0) | 6 (13.3) | 1.00 |
| Nausea | 46 (21.3) | 15 (20.3) | 31 (21.8) | 0.79 | 11 (16.2) | 2 (8.7) | 9 (20.0) | 0.31 |
| Transaminitis | 1 (0.5) | 1 (1.3) | 0 (0.0) | 0.34 | 0 (0.0) | 0 (0.0) | 0 (0.0) | NA |
| Vomiting | 15 (6.9) | 5 (6.8) | 10 (7.0) | 0.94 | 13 (19.1) | 5 (21.7) | 8 (17.8) | 0.69 |
|  |  |  |  |  |  |  |  |  |
| **Musculoskeletal** | 105 (48.6) | 28 (37.8) | 77 (54.2) | **0.02** | 31 (45.6) | 9 (39.1) | 21 (46.7) | 0.35 |
| Muscle/body aches | 105 (48.6) | 28 (37.8) | 77 (54.2) | **0.02** | 30 (44.1) | 9 (39.1) | 21 (46.7) | 0.45 |
| Red or bruised toes | 2 (0.9) | 1 (1.3) | 1 (0.7) | 1.00 | 0 (0.0) | 0 (0.0) | 0 (0.0) | NA |
| Rash | 1 (0.5) | 0 (0.0) | 1 (0.7) | 1.00 | 1 (1.5) | 0 (0.0) | 1 (2.2) | 1.00 |
|  |  |  |  |  |  |  |  |  |
| **Other^d^** | 11 (5.1) | 4 (5.4) | 7 (4.9) | 1.00 | 0 (0.0) | 0 (0.0) | 0 (0.0) | NA |
| **≥6 symptoms** | 127 (58.8) | 42 (56.8) | 85 (59.9) | 0.66 | 29 (42.6) | 6 (26.1) | 23 (51.1) | **0.05^g^** |
| Note: Totals for all individual symptoms (e.g., fever, malaise) per category (e.g., systemic) may not equal category total because categories were defined as at least one of all individual symptoms (i.e., a participant with fever, chills/rigors, and malaise would contribute the same to the systemic total as a participant with only fever). **Boldface** indicates statistical significance. | | | | | | | | |
| ^a^Four asymptomatic outpatient adults and 7 asymptomatic outpatient children excluded from table. | | | | | | | | |
| ^b^Differences in proportions estimated using X^2^ or Fischer’s exact test when appropriate. | | | | | | | | |
| ^c^Fatigue and/or tiredness includes “abnormally sleepy”, “fatigue”, “lethargy”, and “tiredness” self-reported and/or documented in the electronic health record. | | | | | | | | |
| ^d^Other included the following: red bump on left cheek; blurred vision, watery eyes; intermittent burning sensation in hands and feet; sweats; myalgia, possibly due to arthritis and not COVID-19; feels dehydrated; mild metabolic acidosis; tooth pain; “barely audible” expiration diffusion; allergies; thirsty. | | | | | | | | |
| ^e^Rounded value. P-value=0.049. | | | | | | | | |
| ^f^Rounded value. P-value=0.047. | | | | | | | | |
| ^g^Rounded value. P-value=0.048. | | | | | | | | |
