# Supplementary material for: Clinical characterization of acute COVID-19 and Post-COVID-19 Conditions 3 months following infection: A cohort study among Indigenous adults and children in the Southwestern United States
Source: PLOS Glob Public Health. 2025 Mar 18;5(3):e0004204. doi: 10.1371/journal.pgph.0004204 (PMC11918431; doi:10.1371/journal.pgph.0004204)
Supplement: S5 Table — (DOCX) [file pgph.0004204.s006.docx]

| **S5 Table. Characteristics of adult participants with 3-month follow up data, overall and by variant period** | | | | |
| --- | --- | --- | --- | --- |
|  | **Adults (N=216)** | **Pre-Omicron (N=71)** | **Omicron (N=145)** | **p-value^a^** |
|  | **n (%)** | **n (%)** | **n (%)** |  |
| **Median age in years (IQR)** | 47.3 (35.1-58.8) | 44.3 (33.2-55.8) | 48.9 (36.6-59.5) | 0.11 |
| **Age group (in years)** |  |  |  |  |
| 18 to 49 | 120 (55.6) | 46 (64.8) | 74 (51.0) | 0.14 |
| 50 to 64 | 69 (31.9) | 19 (26.8) | 50 (34.5) |  |
| ≥65 | 27 (12.5) | 6 (8.5) | 21 (14.5) |  |
| **Female sex** | 161 (74.5) | 53 (74.7) | 108 (74.5) | 0.98 |
| **Enrollment site (service unit)** |  |  |  |  |
| Chinle | 135 (62.8) | 54 (76.1) | 81 (56.3) | **<0.01** |
| Tuba City | 41 (19.1) | 0 (0.0) | 41 (28.5) |  |
| Whiteriver | 39 (18.1) | 17 (23.9) | 22 (15.3) |  |
| **Running water in home^b^** | 177 (81.9) | 58 (81.7) | 119 (82.1) | 0.70 |
| **Wood used to heat home** | 139 (64.4) | 47 (66.2) | 92 (63.5) | 0.69 |
| **Education level^b,c^** |  |  |  |  |
| Some high school or less | 17 (8.3) | 7 (10.8) | 10 (7.1) | 0.84 |
| High school diploma or GED | 73 (35.4) | 22 (33.9) | 51 (36.2) |  |
| Some college or AA | 92 (44.7) | 29 (44.6) | 63 (44.7) |  |
| Completed degree, including graduate | 24 (11.7) | 7 (10.8) | 17 (12.1) |  |
| **Medical condition or COVID-19 risk factor present** |  |  |  |  |
| Alcohol and/or substance abuse | 19 (8.8) | 8 (11.3) | 11 (7.6) | 0.37 |
| Asthma | 33 (15.3) | 7 (9.9) | 26 (17.9) | 0.12 |
| Chronic lung disease | 6 (2.8) | 1 (1.4) | 5 (3.5) | 0.67 |
| Current or former smoker | 36 (17.1) | 11 (15.9) | 25 (17.6) | 0.76 |
| Chronic kidney disease | 5 (2.3) | 2 (2.8) | 3 (2.1) | 0.67 |
| Chronic liver disease | 9 (4.2) | 3 (4.2) | 6 (4.1) | 1.00 |
| Diabetes (type 1 or 2) | 72 (33.3) | 26 (36.6) | 46 (31.7) | 0.47 |
| Heart condition, excluding hypertension | 17 (7.9) | 4 (5.6) | 13 (9.0) | 0.59 |
| Hypertension | 56 (25.9) | 18 (25.4) | 38 (26.2) | 0.89 |
| Immunocompromised | 2 (0.9) | 1 (1.4) | 1 (0.7) | 0.60 |
| Mental health condition | 47 (21.8) | 15 (21.1) | 32 (22.1) | 0.88 |
| Obesity | 67 (31.0) | 21 (29.6) | 46 (31.7) | 0.74 |
| Supplemental oxygen use at home^b^ | 6 (2.3) | 3 (4.2) | 3 (2.1) | 0.40 |
| **COVID-19 vaccination status at time of acute illness^c^** |  |  |  |  |
| Unvaccinated | 31 (14.4) | 17 (23.9) | 14 (9.7) | **<0.01** |
| Completed primary series only | 129 (59.7) | 54 (76.1) | 75 (51.7) |  |
| Completed primary series + ≥1 booster dose | 56 (25.9) | 0 (0.0) | 56 (38.6) |  |
| **Medical presentation for acute illness** |  |  |  |  |
| Outpatient^d^ | 194 (89.8) | 64 (90.1) | 130 (89.7) | 0.91 |
| Inpatient | 22 (10.2) | 7 (9.9) | 15 (10.3) |  |
| **Symptomatic acute illness** | 212 (98.2) | 70 (98.6) | 142 (97.9) | 0.74 |
| **Self-reported or serologic evidence of prior SARS-CoV-2 infection^e^** |  |  |  |  |
| No | 63 (58.3) | 28 (75.7) | 35 (49.3) | **0.01** |
| Yes | 45 (41.7) | 9 (24.2) | 36 (50.7) |  |
| **Received any treatment for acute illness** |  |  |  |  |
| No | 118 (54.6) | 11 (15.5) | 107 (73.8) | **<0.01** |
| Yes | 98 (45.4) | 60 (84.5) | 38 (26.2) |  |
| **Received monoclonal antibodies to treat acute illness** |  |  |  |  |
| No | 138 (63.9) | 16 (22.5) | 122 (84.1) | **<0.01** |
| Yes | 78 (36.1) | 55 (77.5) | 23 (15.9) |  |
| **Received antivirals to treat acute illness** |  |  |  |  |
| No | 164 (75.9) | 62 (87.3) | 102 (70.3) | **0.01** |
| Yes | 52 (24.1) | 9 (12.7) | 43 (29.7) |  |
| **Post-COVID-19 condition** |  |  |  |  |
| No | 131 (60.7) | 45 (63.4) | 86 (59.3) | 0.57 |
| Yes | 86 (39.8) | 26 (36.6) | 59 (40.7) |  |
| AA, Associate of Arts degree; GED, General Educational Development; IQR, interquartile range. | | | | |
| Note: Variant predominance was defined as the period during which Omicron was detected in >50% of sequenced cases using national trends,^22^ and corroborated by viral genomes sequenced as part of the current study. Omicron was the predominant variant from December 25, 2021 onwards. **Boldface** indicates statistical significance. | | | | |
| ^a^Differences in proportions for categorical variables estimated using Pearson *Χ*^2^ test or Fischer’s exact test when appropriate. | | | | |
| ^b^10 (4.6%) participants missing education level; 3 (1.4%) missing running water in home; 1 (0.5%) missing home oxygen use. | | | | |
| ^c^Completed primary series = Received at least two doses of an approved mRNA COVID-19 vaccine primary series or one dose of an approved non-mRNA vaccine ≥14 days prior to illness onset. May or may not have received ≥1 booster dose. | | | | |
| ^d^Outpatient = participants enrolled at outpatient clinics, Emergency Departments, or SARS-CoV-2 testing clinics. | | | | |
| ^e^Serologic evidence of prior infection determined by blood specimen positive for nucleocapsid IgG antibody. Only blood specimens collected within 1 week of illness onset included (total n=108). Twelve (10.0%) missing self-reported prior infection and/or serologic evidence. | | | | |
