# Supplementary material for: Clinical characterization of acute COVID-19 and Post-COVID-19 Conditions 3 months following infection: A cohort study among Indigenous adults and children in the Southwestern United States
Source: PLOS Glob Public Health. 2025 Mar 18;5(3):e0004204. doi: 10.1371/journal.pgph.0004204 (PMC11918431; doi:10.1371/journal.pgph.0004204)
Supplement: S6 Table — (DOCX) [file pgph.0004204.s007.docx]

| **S6 Table. Self-reported symptoms three months post-acute illness, by age and medical presentation** | | | | | |
| --- | --- | --- | --- | --- | --- |
|  | **Adults (≥18 years)** | | | **Children (<18 years)** | |
|  | **Total (N=216)** | **Inpatient (n=22)** | **Outpatient (n=194)** | **Total (N=69)^a^** | **Outpatient (n=61)** |
|  | **n (%)** | **n (%)** | **n (%)** | **n (%)** | **n (%)** |
| **Systemic** | 41 (19.0) | 6 (27.3) | 35 (18.0) | 3 (4.3) | 3 (4.9) |
| Chills | 3 (1.4) | 1 (4.5) | 2 (1.0) | 1 (1.4) | 1 (1.6) |
| Fever | 3 (1.4) | 0 (0.0) | 3 (1.5) | 2 (2.9) | 2 (3.3) |
| Hair loss | 1 (0.5) | 1 (4.5) | 0 (0.0) | 0 (0.0) | 0 (0.0) |
| Tiredness^b^ | 24 (11.1) | 5 (22.7) | 19 (9.8) | 1 (1.4) | 1 (1.6) |
| Weak/dizzy | 11 (5.1) | 1 (4.5) | 10 (5.2) | 2 (2.9) | 2 (3.3) |
|  |  |  |  |  |  |
| **Respiratory** | 33 (15.3) | 9 (40.9) | 24 (12.4) | 7 (10.1) | 6 (9.8) |
| Chest pain | 2 (0.9) | 1 (4.5) | 1 (0.5) | 0 (0.0) | 0 (0.0) |
| Cough | 20 (9.3) | 6 (27.3) | 14 (7.2) | 7 (10.1) | 6 (9.8) |
| Shortness of breath^c^ | 8 (3.7) | 4 (18.2) | 4 (2.1) | 0 (0.0) | 0 (0.0) |
| Sputum production | 11 (5.1) | 3 (13.6) | 8 (4.1) | 2 (2.9) | 2 (3.3) |
| Wheeze | 10 (4.6) | 3 (13.6) | 7 (3.6) | 2 (2.9) | 2 (3.3) |
|  |  |  |  |  |  |
| **Head, ear, nose, throat** | 40 (18.5) | 4 (18.2) | 36 (18.6) | 6 (8.7) | 6 (9.8) |
| Congestion | 2 (0.9) | 0 (0.0) | 2 (1.0) | 1 (1.4) | 1 (1.6) |
| Conjunctivitis | 4 (1.9) | 1 (4.5) | 3 (1.5) | 1 (1.4) | 1 (1.6) |
| Headache | 26 (12.0) | 3 (13.6) | 23 (11.9) | 2 (2.9) | 2 (3.3) |
| Runny nose | 13 (6.0) | 1 (4.5) | 12 (6.2) | 4 (5.8) | 4 (6.6) |
| Sore throat | 7 (3.2) | 2 (9.1) | 5 (2.6) | 2 (2.9) | 2 (3.3) |
|  |  |  |  |  |  |
| **Neurologic** | 4 (1.9) | 2 (9.1) | 2 (1.0) | 2 (2.9) | 2 (3.3) |
| Loss of taste or smell | 4 (1.9) | 2 (9.1) | 2 (1.0) | 2 (2.9) | 2 (3.3) |
|  |  |  |  |  |  |
| **Mental/behavioral** | 7 (3.2) | 3 (13.6) | 4 (2.1) | 0 (0.0) | 0 (0.0) |
| Anxiety | 1 (0.5) | 0 (0.0) | 1 (0.5) | 0 (0.0) | 0 (0.0) |
| Confusion | 6 (2.8) | 3 (13.6) | 3 (1.5) | 0 (0.0) | 0 (0.0) |
|  |  |  |  |  |  |
| **Gastrointestinal** | 15 (6.9) | 5 (22.7) | 10 (5.2) | 2 (2.9) | 2 (3.3) |
| Abdominal pain | 6 (2.8) | 2 (9.1) | 4 (2.1) | 0 (0.0) | 0 (0.0) |
| Diarrhea | 2 (0.9) | 1 (4.5) | 1 (0.5) | 2 (2.9) | 2 (3.3) |
| Loss of appetite | 7 (3.2) | 4 (18.2) | 3 (1.5) | 1 (1.4) | 1 (1.6) |
| Nausea | 6 (2.8) | 1 (4.5) | 5 (2.6) | 1 (1.4) | 1 (1.6) |
| Vomiting | 0 (0.0) | 0 (0.0) | 0 (0.0) | 1 (1.4) | 1 (1.6) |
|  |  |  |  |  |  |
| **Musculoskeletal** | 16 (7.4) | 2 (9.1) | 14 (7.2) | 0 (0.0) | 0 (0.0) |
| Myalgia | 16 (7.4) | 2 (9.1) | 14 (7.2) | 0 (0.0) | 0 (0.0) |
| Red or bruised toes | 0 (0.0) | 0 (0.0) | 0 (0.0) | 0 (0.0) | 0 (0.0) |
|  |  |  |  |  |  |
| **Other^d^** | 1 (0.5) | 1 (4.5) | 0 (0.0) | 0 (0.0) | 0 (0.0) |
| **Any symptom** | 73 (33.8) | 12 (54.6) | 61 (31.4) | 9 (13.0) | 8 (13.1) |
|  |  |  |  |  |  |
| **1 symptom** | 29 (13.4) | 5 (22.7) | 24 (12.4) | 3 (4.4) | 2 (3.3) |
| **2 symptoms** | 19 (26.4) | 1 (4.6) | 18 (9.3) | 3 (4.4) | 3 (4.9) |
| **≥3 symptoms** | 25 (34.7) | 6 (27.3) | 19 (9.8) | 3 (4.4) | 3 (4.9) |
| Note: Self-reported symptoms may differ from signs and symptoms recorded from the EHR and may differ from Figure 1 in the main document. Thirteen adults and two children did not have any self-reported symptoms and were classified as having PCC based on signs and symptoms or a new diagnosis noted in the EHR. | | | | | |
| ^a^Data on inpatient children not presented because of sparse data (N<10) | | | | | |
| ^b^Includes lethargy and abnormally sleepy | | | | | |
| ^c^Includes “rapid or shallow breathing” | | | | | |
| ^d^Other includes: foot pain near toes | | | | | |
