# Supplementary material for: Clinical characterization of acute COVID-19 and Post-COVID-19 Conditions 3 months following infection: A cohort study among Indigenous adults and children in the Southwestern United States
Source: PLOS Glob Public Health. 2025 Mar 18;5(3):e0004204. doi: 10.1371/journal.pgph.0004204 (PMC11918431; doi:10.1371/journal.pgph.0004204)
Supplement: S7 Table — (DOCX) [file pgph.0004204.s008.docx]

| **S7 Table. Signs and symptoms recorded in EHR three months post-acute illness, by age and medical presentation** | | | | | |
| --- | --- | --- | --- | --- | --- |
|  | **Adults (≥18 years)** | | | **Children (<18 years)** | |
|  | **Total (N=216)** | **Inpatient (n=22)** | **Outpatient (n=194)** | **Total (N=69)^a^** | **Outpatient (n=61)** |
|  | **n (%)** | **n (%)** | **n (%)** | **n (%)** | **n (%)** |
| **Systemic** | 9 (4.2) | 3 (13.6) | 6 (3.1) | 0 (0.0) | 0 (0.0) |
| Dizziness on standing | 2 (0.9) | 0 (0.0) | 2 (1.0) | 0 (0.0) | 0 (0.0) |
| Fatigue/tiredness | 5 (2.3) | 2 (9.1) | 3 (1.5) | 0 (0.0) | 0 (0.0) |
| Fever | 0 (0.0) | 0 (0.0) | 0 (0.0) | 0 (0.0) | 0 (0.0) |
| MIS-C/A | 0 (0.0) | 0 (0.0) | 0 (0.0) | 0 (0.0) | 0 (0.0) |
| Symptoms that worsen after exertion | 4 (1.9) | 2 (9.1) | 2 (1.0) | 0 (0.0) | 0 (0.0) |
|  |  |  |  |  |  |
| **Respiratory** | 16 (7.4) | 5 (22.7) | 9 (4.6) | 1 (1.4) | 0 (0.0) |
| Bronchiectasis | 0 (0.0) | 0 (0.0) | 0 (0.0) | 0 (0.0) | 0 (0.0) |
| Chronic bronchitis | 0 (0.0) | 0 (0.0) | 0 (0.0) | 0 (0.0) | 0 (0.0) |
| Chest pain | 3 (1.4) | 2 (9.1) | 1 (0.5) | 0 (0.0) | 0 (0.0) |
| Cough | 7 (3.2) | 1 (4.5) | 6 (3.1) | 1 (1.4) | 0 (0.0) |
| Home oxygen supplementation | 5 (2.3) | 4 (18.2) | 1 (0.5) | 0 (0.0) | 0 (0.0) |
| Pneumonia | 1 (0.5) | 0 (0.0) | 1 (0.5) | 0 (0.0) | 0 (0.0) |
| Shortness of breath | 6 (2.8) | 3 (13.6) | 3 (1.5) | 0 (0.0) | 0 (0.0) |
| Wheeze, asthma | 0 (0.0) | 0 (0.0) | 0 (0.0) | 0 (0.0) | 0 (0.0) |
|  |  |  |  |  |  |
| **Head, ear, nose, throat** | 2 (0.9) | 1 (4.5) | 1 (0.5) | 0 (0.0) | 0 (0.0) |
| Headache | 2 (0.9) | 1 (4.5) | 1 (0.5) | 0 (0.0) | 0 (0.0) |
|  |  |  |  |  |  |
| **Neurologic** | 1 (0.5) | 0 (0.0) | 1 (0.5) | 0 (0.0) | 0 (0.0) |
| Loss of taste or smell | 1 (0.5) | 0 (0.0) | 1 (0.5) | 0 (0.0) | 0 (0.0) |
|  |  |  |  |  |  |
| **Mental** | 3 (1.4) | 1 (4.5) | 2 (1.0) | 1 (1.4) | 1 (1.6) |
| Anxiety | 2 (0.9) | 0 (0.0) | 2 (1.0) | 1 (1.4) | 1 (1.6) |
| Brain fog | 0 (0.0) | 0 (0.0) | 0 (0.0) | 0 (0.0) | 0 (0.0) |
| Depression | 2 (0.9) | 1 (4.5) | 1 (0.5) | 0 (0.0) | 0 (0.0) |
|  |  |  |  |  |  |
| **Cardiac** | 1 (0.5) | 0 (0.0) | 1 (0.5) | 0 (0.0) | 0 (0.0) |
| Cardiomyopathy | 0 (0.0) | 0 (0.0) | 0 (0.0) | 0 (0.0) | 0 (0.0) |
| Heart palpitations | 1 (0.5) | 0 (0.0) | 1 (0.5) | 0 (0.0) | 0 (0.0) |
| Heart inflammation | 0 (0.0) | 0 (0.0) | 0 (0.0) | 0 (0.0) | 0 (0.0) |
|  |  |  |  |  |  |
| **Gastrointestinal** | 3 (1.4) | 1 (4.5) | 2 (1.0) | 0 (0.0) | 0 (0.0) |
| Abdominal pain | 2 (0.9) | 1 (4.5) | 1 (0.5) | 0 (0.0) | 0 (0.0) |
| Diarrhea | 0 (0.0) | 0 (0.0) | 0 (0.0) | 0 (0.0) | 0 (0.0) |
| Loss of appetite | 1 (0.5) | 0 (0.0) | 1 (0.5) | 0 (0.0) | 0 (0.0) |
|  |  |  |  |  |  |
| **Musculoskeletal, dermatologic** | 0 (0.0) | 0 (0.0) | 0 (0.0) | 1 (1.4) | 1 (1.6) |
| Muscle or joint aches | 0 (0.0) | 0 (0.0) | 0 (0.0) | 1 (1.4) | 1 (1.6) |
| Rash | 0 (0.0) | 0 (0.0) | 0 (0.0) | 0 (0.0) | 0 (0.0) |
|  |  |  |  |  |  |
| **Other**^b^ | 5 (2.3) | 1 (4.5) | 4 (2.1) | 0 (0.0) | 0 (0.0) |
| **Any sign or symptom** | 23 (10.7) | 7 (31.8) | 16 (8.3) | 2 (2.9) | 1 (1.6) |
| **1 sign or symptom** | 9 (4.2) | 2 (9.1) | 7 (3.6) | 1 (1.5) | 0 (0.0) |
| **2 signs or symptoms** | 7 (3.2) | 3 (13.6) | 4 (2.1) | 0 (0.0) | 0 (0.0) |
| **≥3 signs or symptoms** | 7 (3.2) | 2 (9.1) | 5 (2.6) | 1 (1.5) | 1 (1.6) |
| EHR, electronic health record; MIS-C/A, Multi-system inflammatory disease in children (C) or adults (A); POTS, Postural orthostatic tachycardia syndrome; PTSD, Post-traumatic stress disorder. | | | | | |
| Note: Signs and symptoms recorded in the EHR may differ from those self-reported and may differ from Figure 1 in the main document. 63 adults and nine children did not have any signs or symptoms noted in the EHR and were classified as having PCC based on self-reported symptoms or a new condition only. | | | | | |
| ^a^Data on inpatient children not presented because of sparse data (N<10) | | | | | |
| ^b^Other include right shin splints; sluggish, fatigue of unknown cause, needs monitoring of thyroid and medication adjustment; lightheadedness; “using gummies to help sleep,” sometimes does not want to get out of bed; protein C deficiency. | | | | | |
