# Supplementary material for: Clinical characterization of acute COVID-19 and Post-COVID-19 Conditions 3 months following infection: A cohort study among Indigenous adults and children in the Southwestern United States
Source: PLOS Glob Public Health. 2025 Mar 18;5(3):e0004204. doi: 10.1371/journal.pgph.0004204 (PMC11918431; doi:10.1371/journal.pgph.0004204)
Supplement: S8 Table — (DOCX) [file pgph.0004204.s009.docx]

| **S8 Table. New conditions recorded in EHR three months post-acute illness, by age and medical presentation** | | | | | |
| --- | --- | --- | --- | --- | --- |
|  | **Adults (≥18 years)** | | | **Children (<18 years)** | |
|  | **Total (N=216)** | **Inpatient (n=22)** | **Outpatient (n=194)** | **Total (N=69)^a^** | **Outpatient (n=61)** |
|  | **n (%)** | **n (%)** | **n (%)** | **n (%)** | **n (%)** |
| **Mental health** |  |  |  |  |  |
| Anxiety^b^ | 0 (0.0) | 0 (0.0) | 0 (0.0) | 0 (0.0) | 0 (0.0) |
| Depression^b^ | 2 (0.9) | 1 (4.5) | 1 (0.5) | 0 (0.0) | 0 (0.0) |
| PTSD | 0 (0.0) | 0 (0.0) | 0 (0.0) | 0 (0.0) | 0 (0.0) |
| Other mental conditions | 1 (0.5) | 0 (0.0) | 1 (0.5) | 1 (1.5) | 1 (1.6) |
| Sleep disorder | 2 (0.9) | 1 (4.5) | 1 (0.5) | 0 (0.0) | 0 (0.0) |
| Substance abuse | 1 (0.5) | 1 (4.6) | 0 (0.0) | 0 (0.0) | 0 (0.0) |
|  |  |  |  |  |  |
| **Neurologic** |  |  |  |  |  |
| GBS | 0 (0.0) | 0 (0.0) | 0 (0.0) | 0 (0.0) | 0 (0.0) |
| Neuromuscular disorders | 0 (0.0) | 0 (0.0) | 0 (0.0) | 0 (0.0) | 0 (0.0) |
| Seizure | 0 (0.0) | 0 (0.0) | 0 (0.0) | 0 (0.0) | 0 (0.0) |
|  |  |  |  |  |  |
| **Cardiovascular** |  |  |  |  |  |
| Cardiovascular disease | 1 (0.5) | 0 (0.0) | 1 (0.5) | 0 (0.0) | 0 (0.0) |
| POTS | 0 (0.0) | 0 (0.0) | 0 (0.0) | 0 (0.0) | 0 (0.0) |
|  |  |  |  |  |  |
| **Gastrointestinal** |  |  |  |  |  |
| Gastritis | 0 (0.0) | 0 (0.0) | 0 (0.0) | 0 (0.0) | 0 (0.0) |
| GERD | 1 (0.5) | 0 (0.0) | 1 (0.5) | 0 (0.0) | 0 (0.0) |
|  |  |  |  |  |  |
| **Renal** |  |  |  |  |  |
| Chronic kidney disease | 0 (0.0) | 0 (0.0) | 0 (0.0) | 0 (0.0) | 0 (0.0) |
| Dialysis | 0 (0.0) | 0 (0.0) | 0 (0.0) | 0 (0.0) | 0 (0.0) |
| Kidney damage | 0 (0.0) | 0 (0.0) | 0 (0.0) | 0 (0.0) | 0 (0.0) |
|  |  |  |  |  |  |
| **Hemolytic, Vascular** |  |  |  |  |  |
| Coagulation and hemorrhagic event | 0 (0.0) | 0 (0.0) | 0 (0.0) | 0 (0.0) | 0 (0.0) |
|  |  |  |  |  |  |
| **Endocrine** |  |  |  |  |  |
| Diabetes type 1 or 2 | 0 (0.0) | 0 (0.0) | 0 (0.0) | 0 (0.0) | 0 (0.0) |
|  |  |  |  |  |  |
| **Immunological** |  |  |  |  |  |
| Autoimmune | 0 (0.0) | 0 (0.0) | 0 (0.0) | 0 (0.0) | 0 (0.0) |
|  |  |  |  |  |  |
| **Any new condition** | 8 (3.7) | 2 (9.1) | 6 (3.1) | 1 (1.5) | 1 (1.6) |
| **1 new condition** | 7 (3.2) | 1 (4.6) | 6 (3.1) | 1 (1.5) | 1 (1.6) |
| **2 new conditions** | 1 (0.5) | 1 (4.6) | 0 (0.0) | 0 (0.0) | 0 (0.0) |
| EHR electronic health record; GBS, Guillain-Barré syndrome; GERD, Gastroesophageal reflux disease; POTS, Postural tachycardia syndrome; PTSD, Post-traumatic stress disorder | | | | | |
| Note: 78 adults and nine children did not have any new conditions noted in the EHR and were classified as having PCC based on self-reported symptoms or signs or symptoms in the EHR only. | | | | | |
| ^a^Data on inpatient children not presented because of sparse data (N<10) | | | | | |
| ^b^Anxiety and depression differ here from anxiety and depression reported as “signs and symptoms” in that the former are recorded as diagnosed by a healthcare provider, while the latter may not be based on a diagnosis. 1 adult reported depression as a “sign or symptom” (S6 Table ) and had depression recorded as diagnosed. | | | | | |
