# Supplementary material for: Clinical characterization of acute COVID-19 and Post-COVID-19 Conditions 3 months following infection: A cohort study among Indigenous adults and children in the Southwestern United States
Source: PLOS Glob Public Health. 2025 Mar 18;5(3):e0004204. doi: 10.1371/journal.pgph.0004204 (PMC11918431; doi:10.1371/journal.pgph.0004204)
Supplement: S9 Table — (DOCX) [file pgph.0004204.s010.docx]

| **S9 Table. Selected underlying medical conditions associated with PCC three months post-acute illness among adults enrolled during Omicron predominance** | | | | | |
| --- | --- | --- | --- | --- | --- |
|  | **Total (N=145)** | | **With PCC (n=60)** | **P-value^b^** | **RR (95% CI)** |
|  | **n** | | **n (%)^a^** |  |  |
| **Alcohol and/or substance abuse** |  | |  |  |  |
| No | 134 | | 56 (41.8) | 0.73 | REF |
| Yes | 11 | | 4 (36.4) |  | 0.87 (0.39-1.96) |
| **Asthma^c^** |  | |  |  |  |
| No | 119 | | 45 (37.8) | 0.06 | REF |
| Yes | 26 | | 15 (57.7) |  | **1.53 (1.02-2.28)** |
| **Anxiety^c^** |  |  | |  |  |
| No | 136 | 56 (41.2) | | 0.85 | REF |
| Yes | 9 | 4 (44.4) | |  | 1.08 (0.50-2.31) |
| **Chronic lung disease^c^** |  | |  |  |  |
| No | 140 | | 59 (42.1) | 0.32 | REF |
| Yes | 5 | | 1 (20.0) |  | 0.47 (0.08-2.79) |
| **Chronic kidney disease** |  | |  |  |  |
| No | 142 | | 58 (40.9) | 0.37 | REF |
| Yes | 3 | | 2 (66.7) |  | 1.63 (0.71-3.73) |
| **Chronic liver disease** |  | |  |  |  |
| No | 139 | | 57 (41.0) | 0.66 | REF |
| Yes | 6 | | 3 (50.0) |  | 1.22 (0.53-2.79) |
| **Current or former smoker** |  | |  |  |  |
| No | 117 | | 51 (43.6) | 0.29 | REF |
| Yes | 25 | | 8 (32.0) |  | 0.73 (0.40-1.35) |
| **Diabetes (type 1 or 2)^c^** |  | |  |  |  |
| No | 99 | | 37 (37.4) | 0.15 | REF |
| Yes | 46 | | 23 (50.0) |  | 1.34 (0.91-1.97) |
| **Depression^c^** |  |  | |  |  |
| No | 121 | 48 (39.7) | | 0.35 | REF |
| Yes | 24 | 12 (50.0) | |  | 1.26 (0.78-2.00) |
| **Heart condition, excluding hypertension^c^** |  | |  |  |  |
| No | 132 | | 54 (40.9) | 0.71 | REF |
| Yes | 13 | | 6 (46.2) |  | 1.13 (0.60-2.11) |
| **Hypertension^c^** |  | |  |  |  |
| No | 107 | | 38 (35.5) | **0.02** | REF |
| Yes | 38 | | 22 (57.9) |  | **1.63 (1.12-2.37)** |
| **Immunocompromised^c^** |  | |  |  |  |
| No | 144 | | 59 (41.0) | 0.23 | REF |
| Yes | 1 | | 1 (100.0) |  | **2.44 (2.00-2.97)** |
| **Obesity^c^** |  | |  |  |  |
| No | 99 | | 41 (41.4) | 0.99 | REF |
| Yes | 46 | | 19 (41.3) |  | 1.00 (0.66-1.51) |
| CI, confidence interval; RR, risk ratio; REF, reference category | | | | | |
| Note: **Boldface** indicates statistical significance (defined as p-value < 0.05 or 95% CI that did not include 1.00). | | | | | |
| ^a^Row percentage indicating proportion that developed PCC among each covariate level. | | | | | |
| ^b^Differences in proportions estimated using Pearson *Χ*^2^ test or Fischer’s exact test when appropriate. | | | | | |
| ^c^Individual comorbidity included in composite “presence of underlying medical condition” used in final multivariable model presented in Table 4 of the main document. | | | | | |
