# Supplementary material for: Clinical characterization of acute COVID-19 and Post-COVID-19 Conditions 3 months following infection: A cohort study among Indigenous adults and children in the Southwestern United States
Source: PLOS Glob Public Health. 2025 Mar 18;5(3):e0004204. doi: 10.1371/journal.pgph.0004204 (PMC11918431; doi:10.1371/journal.pgph.0004204)
Supplement: S10 Table — (DOCX) [file pgph.0004204.s011.docx]

| **S10 Table. Sociodemographic and clinical characteristics associated with PCC at three months post-acute illness among adults enrolled during Omicron predominance – definition of PCC restricted to self-reported symptoms** | | | | | |
| --- | --- | --- | --- | --- | --- |
|  | **Total (N=145)** | **With PCC (n=54)^a^** | **P-value^b^** | **RR (95% CI)** | **aRR (95% CI)** |
|  | **n** | **n (%)** |  |  |  |
| **Sex** |  |  |  |  |  |
| Male | 37 | 10 (27.0) | 0.14 | REF | REF |
| Female | 108 | 44 (40.7) |  | 1.51 (0.85-2.69) | **1.79 (1.03-3.09)** |
| **Age group (in years)** |  |  |  |  |  |
| 18–49 | 74 | 22 (29.7) | 0.16 | REF | REF |
| 50–64 | 50 | 23 (46.0) |  | 1.55 (0.97-2.46) | 1.48 (0.93-2.36) |
| ≥65 | 21 | 9 (42.9) |  | 1.44 (0.79-2.65) | 1.35 (0.78-2.33) |
| **Presence of underlying medical condition** |  |  |  |  |  |
| None | 42 | 12 (28.6) | 0.17 | REF | REF |
| ≥1^c^ | 103 | 42 (40.8) |  | 1.43 (0.84-2.43) | 1.32 (0.78-2.25) |
| **Medical presentation at time of acute illness^d^** |  |  |  |  |  |
| Outpatient | 130 | 45 (34.6) | 0.05 | REF | REF |
| Inpatient | 15 | 9 (60.0) |  | **1.73 (1.08-2.79)** | 1.49 (0.93-2.38) |
| **Vaccination status at time of acute illness** |  |  |  |  |  |
| Unvaccinated | 14 | 9 (64.3) | **0.03** | REF | REF |
| Completed primary series^e^ | 131 | 45 (34.4) |  | **0.53 (0.34-0.84)** | **0.47 (0.28-0.78)** |
| CI, confidence interval; PCC, Post-COVID Conditions; RR, risk ratio; aRR, adjusted risk ratio; REF, reference category | | | | | |
| Note: This sensitivity analysis was restricted to adult participants who were enrolled during Omicron variant predominance and completed a 3-month interview; PCC was defined based only on self-reported symptoms only. Omicron variant predominance was defined as the period during which Omicron was detected in >50% of sequenced cases using national trends,^22^ and occurred from December 25, 2021 onwards. Children were excluded from the risk factor analysis because of small sample size (n=11 with PCC). **Boldface** indicates statistical significance (defined as p-value < 0.05 or 95% CI that did not include 1.00). | | | | | |
| ^a^Row percentage indicating proportion that developed PCC among each covariate level. | | | | | |
| ^b^Differences in proportions for categorical variables estimated using Pearson *Χ*^2^ test or Fischer’s exact test when appropriate. | | | | | |
| ^c^Indicates participant has at least one of the following listed in their medical record: anxiety, asthma, chronic lung disease, depression, diabetes type 1 or 2, heart conditions (excluding hypertension only, included [but not limited to] atherosclerotic cardiovascular disease, cardiomyopathy, congestive heart failure, and/or coronary artery disease), hypertension, immunosuppression (due to treatment/therapy, bone marrow or solid organ transplant recipient), and/or obesity. | | | | | |
| ^d^Outpatient = participants enrolled at outpatient clinics, Emergency Departments, or SARS-CoV-2 testing clinics. | | | | | |
| ^e^Completed primary series = Received two doses of an approved mRNA COVID-19 vaccine primary series or one dose of an approved non-mRNA vaccine ≥14 days prior to illness onset. May or may not have received ≥1 booster dose. | | | | | |
