# Supplementary figures and images for: Clinical characterization of acute COVID-19 and Post-COVID-19 Conditions 3 months following infection: A cohort study among Indigenous adults and children in the Southwestern United States
Source: PLOS Glob Public Health. 2025 Mar 18;5(3):e0004204. doi: 10.1371/journal.pgph.0004204 (PMC11918431; doi:10.1371/journal.pgph.0004204)

| **S1 Fig. Flow diagram of participant enrollment and completed study activities** |
| --- |
| 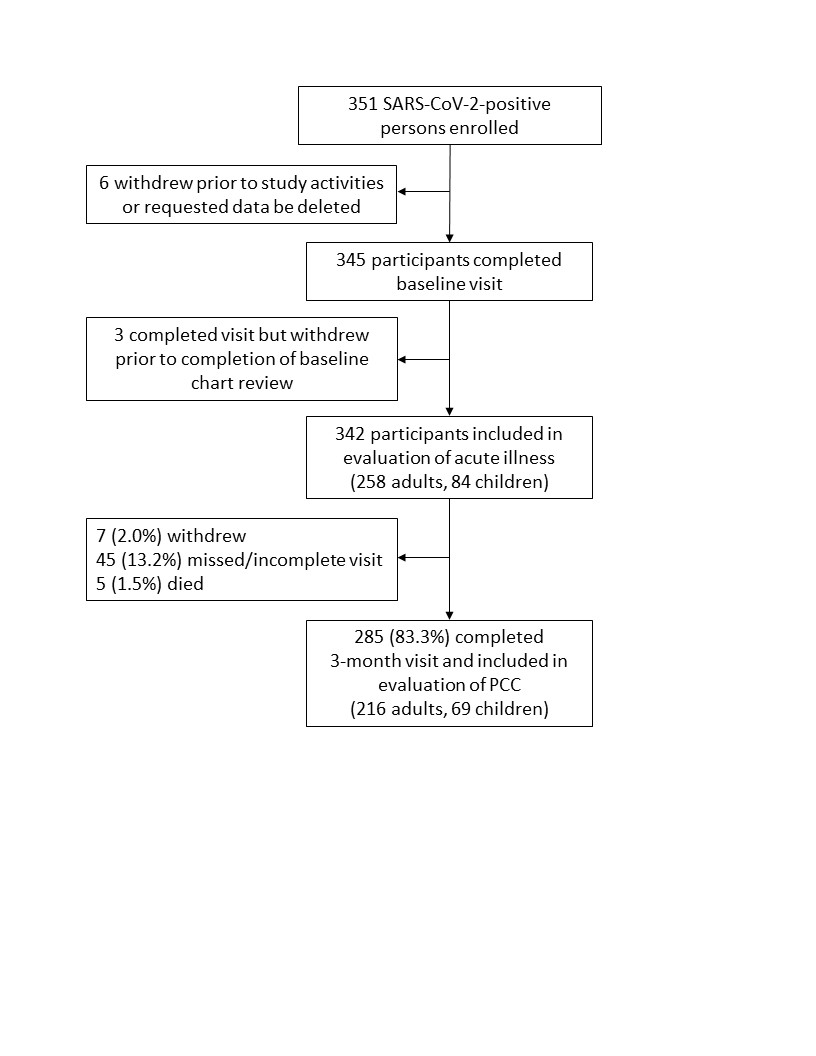 |

Supplement: S1 Fig — (DOCX) [file pgph.0004204.s012.docx]
