# Supplementary material for: Clinical characterization of acute COVID-19 and Post-COVID-19 Conditions 3 months following infection: A cohort study among Indigenous adults and children in the Southwestern United States
Source: PLOS Glob Public Health. 2025 Mar 18;5(3):e0004204. doi: 10.1371/journal.pgph.0004204 (PMC11918431; doi:10.1371/journal.pgph.0004204)
Supplement: S2 Fig — (DOCX) [file pgph.0004204.s013.docx]

| **S2 Figure. Antibody positivity among participants who had a blood specimen collected <1 week after onset of acute illness, by age group, COVID-19 vaccination status, and target protein** | |
| --- | --- |
| **A. Antibody to spike glycoprotein, adults** | **B. Antibody to spike glycoprotein, children** |
|  |  |
| **C. Antibody to Receptor binding domain (RBD), adults** | **D. Antibody to Receptor binding domain (RBD), children** |
|  |  |
| **E. Antibody to nucleocapsid, adults** | **F. Antibody to nucelocapsid, children** |
|  |  |
