# Supplementary material for: Clinical characterization of acute COVID-19 and Post-COVID-19 Conditions 3 months following infection: A cohort study among Indigenous adults and children in the Southwestern United States
Source: PLOS Glob Public Health. 2025 Mar 18;5(3):e0004204. doi: 10.1371/journal.pgph.0004204 (PMC11918431; doi:10.1371/journal.pgph.0004204)
Supplement: S3 Fig — (DOCX) [file pgph.0004204.s014.docx]

| **S3 Figure . SARS-CoV-2 sequencing results for specimens collected during acute illness, by month of swab collection** |  |
| --- | --- |
|  | **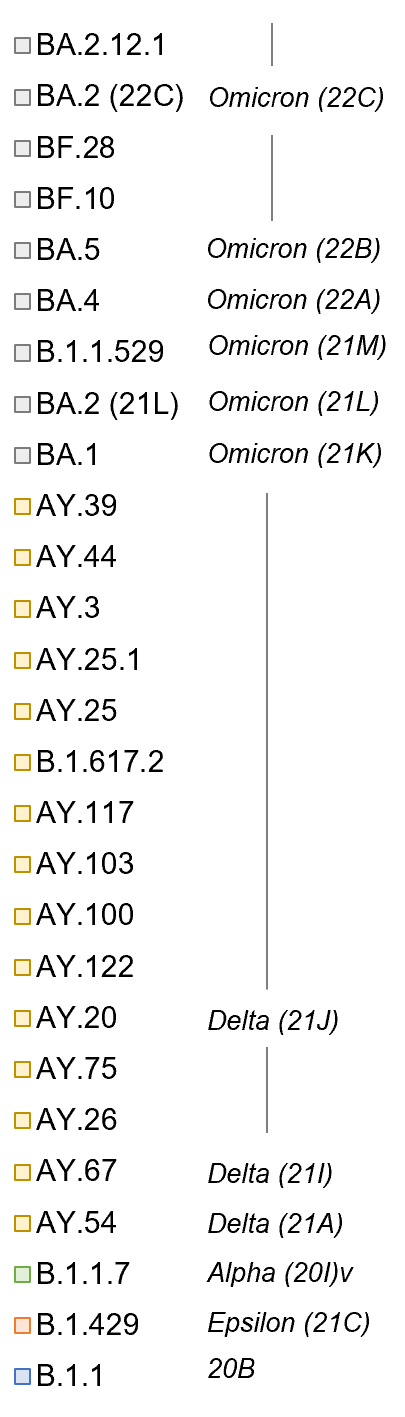** |
